# Single-cell temporal analysis of natural dengue infection reveals skin-homing lymphocyte expansion one day before defervescence

**DOI:** 10.1016/j.isci.2022.104034

**Published:** 2022-03-05

**Authors:** Jantarika Kumar Arora, Anunya Opasawatchai, Tiraput Poonpanichakul, Natnicha Jiravejchakul, Waradon Sungnak, DENFREE Thailand, Anavaj Sakuntabhai, Pratap Singhasivanon, Swangjit Suraamornkul, Tawatchai Yingtaweesak, Khajohnpong Manopwisedjaroen, Nada Pitabut, Oranart Matangkasombut, Sarah A. Teichmann, Ponpan Matangkasombut, Varodom Charoensawan

**Affiliations:** 1 Doctor of Philosophy Program in Biochemistry (International Program), Faculty of Science, Mahidol University, Bangkok 10400, Thailand; 2 Department of Biochemistry, Faculty of Science, Mahidol University, Bangkok 10400, Thailand; 3 Department of Oral Microbiology, Faculty of Dentistry, Mahidol University, Bangkok 10400, Thailand; 4 Integrative Computational Bioscience (ICBS) Center, Mahidol University, Nakorn Pathom 73170, Thailand; 5 Department of Microbiology, Faculty of Science, Mahidol University, Bangkok 10400, Thailand; 6 Systems Biology of Diseases Research Unit, Faculty of Science Mahidol University, Bangkok 10400, Thailand; 7 Department of Microbiology and Center of Excellence on Oral Microbiology and Immunology, Faculty of Dentistry, Chulalongkorn University, Bangkok 10330, Thailand; 8 Research Laboratory of Biotechnology, Chulabhorn Research Institute, Bangkok 10210, Thailand; 9 Wellcome Sanger Institute, Wellcome Trust Genome Campus, Hinxton, Cambridge CB10 1SA, UK

**Keywords:** Single-cell Omics, Systems biology, Immunology

## Abstract

Effective clinical management of acute dengue virus (DENV) infection relies on the timing of suitable treatments during the disease progression. We analyzed single-cell transcriptomic profiles of the peripheral blood mononuclear cell samples from two DENV patients, collected daily during acute phase and also at convalescence. Key immune cell types demonstrated different dynamic responses over the course of the infection. On the day before defervescence (Day −1), we observed the peak expression of several prominent genes in the adaptive immunological pathways. We also characterized unique effector T cell clusters that expressed skin-homing signature genes at Day −1, whereas upregulation of skin and gut homing genes was also observed in plasma cells and plasmablasts during the febrile period. This work provides an overview of unique molecular dynamics that signify the entry of the critical phase, and the findings could improve the patient management of DENV infection.

## Highlights

• Time-course scRNA-seq reveals immune response dynamics during acute dengue infection
• Rapid transcriptional switching was observed one day before fever subsided (Day -1)
• Skin-homing signatures were observed in specific T cells during the febrile phase
• Expansion of skin-homing CD69^+^ PD-1^+^ T cells at Day -1 was confirmed with FACS

## Graphical abstract

**Figure:**
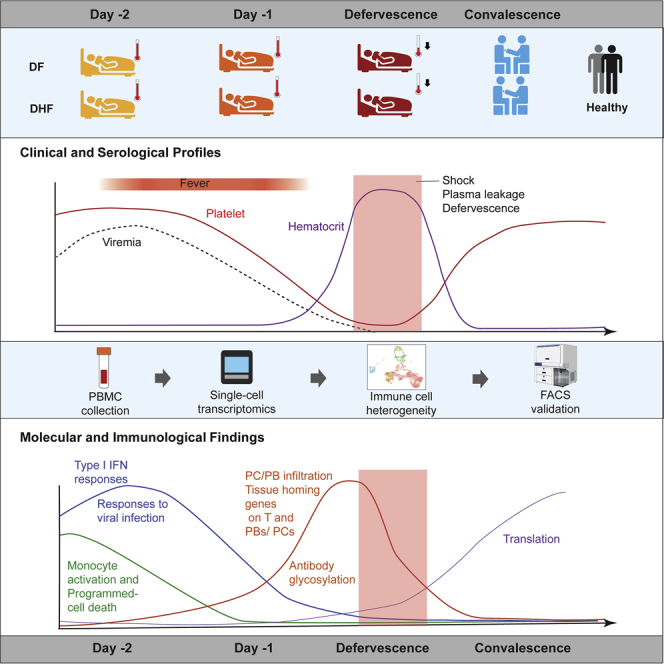

## Introduction

Dengue virus (DENV) is estimated to infect 390 million people worldwide each year (Bhatt et al., 2013). The majority of DENV infections are asymptomatic, however, it was estimated that 96 million patients had apparent symptoms of variable severities annually, making it one of the leading causes of hospitalization in tropical and subtropical countries (Bhatt et al., 2013). Similar to other mosquito-borne diseases, dengue has now emerged in new territories including Europe and North America (Liu-Helmersson et al., 2016; Rivera et al., 2020). DENV infection is clinically classified as mild dengue fever (DF), severe dengue hemorrhagic fever (DHF), and life-threatening dengue shock syndrome (DSS) (WHO Regional Office for South-East Asia, 2011). Current treatments of DENV patients largely rely on supportive care, as there is no specific antiviral therapy, whereas the safety and efficacy of the only licenced live-attenuated tetravalent dengue vaccine (CYD-TDV) are still under debate (Hadinegoro et al., 2015).

The clinical course of DENV infection can be generally divided into three phases: febrile, critical, and convalescent (Kalayanarooj, 2011). The febrile phase usually lasts 2–7 days where both DF and DHF patients present with high-grade fever, malaise, and retro-orbital pains (World Health Organization, 2009). The host immune responses against DENV during this phase are dynamic, dictating the subsequent critical phases. Unlike other acute viral infections, the day the fever subsides, also known as “defervescence”, signifies the start of a very critical phase of 24–48 h. Close patient monitoring is essential as a substantial drop of platelet counts and plasma leakage may occur in DHF, as well as shock in DSS cases (World Health Organization, 2009). The timing of defervescence is, however, difficult to pre-determine accurately, making it challenging to properly triage the patients and plan the fluid management. There is currently still no established molecular marker to help predict the disease progression and plan suitable management for each DENV-infected patient (Kalayanarooj, 2011).

Development of therapeutic and preventive measures of DENV has so far been complicated by not only the complexity of interplaying immune cell types, but also their dynamics against the virus during the course of infection, and these together determine the clinical outcomes in different patients (Diamond and Pierson, 2015; Tsai, W.Y. et al., 2017). The responses of T cells against DENV are highly complex and heterogeneous, and can give rise to detrimental or protective effects (Campos et al., 2018). For example, the cross-reactive T cells to previous heterotypic DENV infection in DHF was found to be less cytotoxic but produce more cytokines than in DF, suggesting their detrimental roles in promoting hemorrhage and shock, so called “T cell original antigenic sin” (Duangchinda et al., 2010; Mongkolsapaya et al., 2003). On the other hand, CD8^+^T cells specific to the DENV immunodominant epitopes have been shown to provide protection against DENV infection in human (Chng et al., 2019) and type I interferon deficient mice model (Elong Ngono et al., 2016; Yauch et al., 2009). For B cells, pre-existing neutralizing antibodies produced by antibody secreting cells (ASCs) (Dejnirattisai et al., 2015), have been shown to shorten the viremic period in secondary DENV patients (Tricou et al., 2011). On the contrary, non-neutralizing antibodies from previous infection with the heterotypic serotype could lead to Fc receptor-mediated viral uptake in a process called antibody-dependent-enhancement (ADE), which could be responsible for the disease pathogenesis (Whitehead et al., 2007).

Earlier examples that showcased the highly dynamic immune systems against DENV infection include the subpopulations of the natural killer (NK) cells, which were activated and peaked during the febrile phase (Keawvichit et al., 2018). Beside the dynamics of immune cell abundances, changes of immunological molecules during the course of DENV infection have also been documented. For instance, several cytokines have been shown to be associated with DENV infection, specifically at the febrile and critical phases (Rathakrishnan et al., 2012), similar to the levels of the platelet activating factor (PAF), which were seen rising and falling by hours before the critical phase (Jeewandara et al., 2015). High-throughput analyses have also been employed to explore the transcriptional signatures associated with the progression and severities in DENV-infected patients, *e.g.,* (Banerjee et al., 2017; Hanley et al., 2021; Popper et al., 2012; Simon-Loriere et al., 2017). However, these transcriptomic studies on host immune responses against DENV so far largely relied on the “bulk” or population-level analyses, which provide average profiles of the entire immune cells, or those that can be sorted by known surface markers. Despite suggestive evidence of dynamic abundances of particular immune cells and expression of key immune genes as possible indicators of the disease progression, there is yet to be a study that comprehensively characterizes the dynamics of all the immune cell types and their underlying molecular biology across the key time points of DENV infection.

To overcome the limitations of the bulk high-throughput analyses, single-cell technology has recently been employed to dissect the responses of different immune cells to the DENV infection, *e.g.,* (Patil et al., 2018; Waickman et al., 2019, 2021; Zanini et al., 2018). Using single-cell RNA-seq (scRNA-seq), Patil et al. have identified a subset of cytotoxic CD4^+^ T cells that were clonally expanded in response to *ex vivo* stimulation with DENV (Patil et al., 2018); whereas Waickman et al. have shown that clonally expanded T cells in response to a DENV vaccine showed unique metabolic changes that signify the effector/memory potential (Waickman et al., 2019). Zanini et al. have profiled the transcriptomes of the virus and host simultaneously, and distinguished the immune responses of the infected cells from the bystanders (Zanini et al., 2018). These studies showcased how single-cell technology provides in-depth insights into the immune responses to the DENV infection at unprecedented single cell levels, but all focused at particular time points or cell types during the course of infection. More recently, for the first time scRNA-seq has been implemented to investigate the transcriptomic patterns of experimental and natural primary DENV-1 infections at multiple time points (Waickman et al., 2021). The study provided insights into common and specific patterns of immune response between the experimental and natural primary infections, however, it did not particularly focus on detailed transcriptional changes on the days leading to the clinically critical period of defervescence.

In this study, we have employed scRNA-seq to exhaustively investigate the dynamics of different immune cell populations in peripheral blood mononuclear cells (PBMCs) and their molecular responses at four time points across the clinical course of DENV infection: two days during the febrile illness (“Day −2”, and “Day −1”), one at defervescence (“Def”), and another at two-week (“Wk2”) convalescence. Based on two adult male patients with secondary DENV-4 infection, one with DF and the other with DHF severities, we have shown that systemic type I interferon responses were elicited early in the febrile illness in the key immune cells, before declining in the convalescences. Remarkably, the most extreme change of the immune cell compositions, and also their transcriptomic profiles occurred one day before the critical period of defervescence (Day −1), as the highest relative proportions of effector T cells and plasma cells were observed. We have also characterized the expression of skin-homing signature genes in clusters of effector CD8^+^ and CD4^+^ T cells, and in ASCs. The protein expression of skin-homing molecules were also validated by flow cytometry in over 20 additional DENV-infected patients and 40 samples, suggesting their potential to relocate to the primary site of the viral entry during the critical phase of the infection.

## Results

### Dynamics of immune cell populations over the clinical course of DENV infection

**Figure 1:**
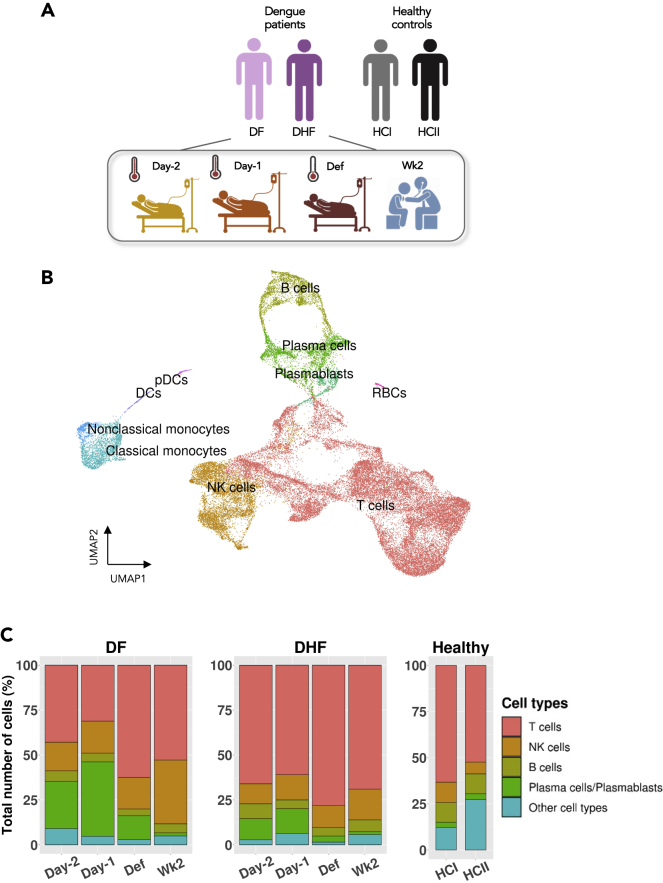
Dynamics of immune cell populations during DENV infection (A) Summary of PBMC samples from two DENV-infected patients with different severities: DF and DHF, across four time points: two days before defervescence (Day −2), one day before defervescence (Day −1), defervescence (Def), and two weeks after defervescence (Wk2). Two healthy PBMCs from independent sources were also included (see Method details). (B) Uniform Mani-fold Approximation and Projection (UMAP) plot showing integrated single-cell PBMC profiles from the two patients and from two healthy controls, colored by cell types. (C) Relative abundances of key immune cell populations in each sample. DF = dengue fever; DHF = dengue hemorrhagic fever; HC = healthy control; DCs = dendritic cells; pDCs = plasmacytoid dendritic cells; and RBCs = red blood cells (see also Figure S1; Tables S1 and S2).

We characterized the overall dynamics of immune cell subpopulations of two male patients with secondary DENV-4 infection, one with DF and the other with DHF, each across four time points during the course of infection, as compared to the two independent healthy controls (HCs), using the integrated scRNAseq profiles from the ten samples and ∼40,000 cells in total (Figures 1A, 1B, and S1; Table S1 and Method details). For both patients, we observed similar overall distributions of immune cell populations, as well as their dynamic patterns across the time points (Figure 1C; Table S2). As expected, T cells were the largest populations in the PBMC samples, and relatively expanded the most at the defervescence (Def) in both DF and DHF (Figure 1C, pink bars).

The next largest populations were plasma cells (PCs) and plasmablasts (PBs), which showed the highest relative abundances during the febrile infection, and peaked one day before defervescence (Day −1) in both patients, but the change was slightly more prominent in DF (Figure 1C, green bars). At the convalescence or two weeks after Def (Wk2), the proportions of PCs and PBs returned to the levels similar to those of HCs, which was considered the baseline level here. For the natural killer (NK) cells (Figure 1C, orange bars), the patterns of their relative abundances across the four time points were less apparent than other cell types, as a slight expansion of NK at Wk2, was only seen in DF, whereas changes of the NK cell abundances in DHF might be confounded by the relative expansion of T cells during the febrile phase, resulting in a lower NK cell proportion. Because of the limited number of patients with different severities, preliminary observations showing the differences between DF and DHF need to be considered with caution. For the rest of this study, we focused on the temporal changes of the immune cells and their expression profiles across the course of infection.

### Time-course transcriptomic profiling reveals key molecular events one day before defervescence

To explore the molecular markers that could potentially be used to indicate specific clinical stages during the narrow window of acute DENV infection, we first asked if and to what extent the changes of overall transcriptomes (*i.e.,* “pseudo-bulk” RNA-seq) could be linked to the clinical manifestations of the disease. Based on the overall correlations of the population-wide transcriptomes, the most diverging transcriptomic patterns as compared to other time points were at Day −1 in both DF and DHF (Figure S2).

**Figure 2:**
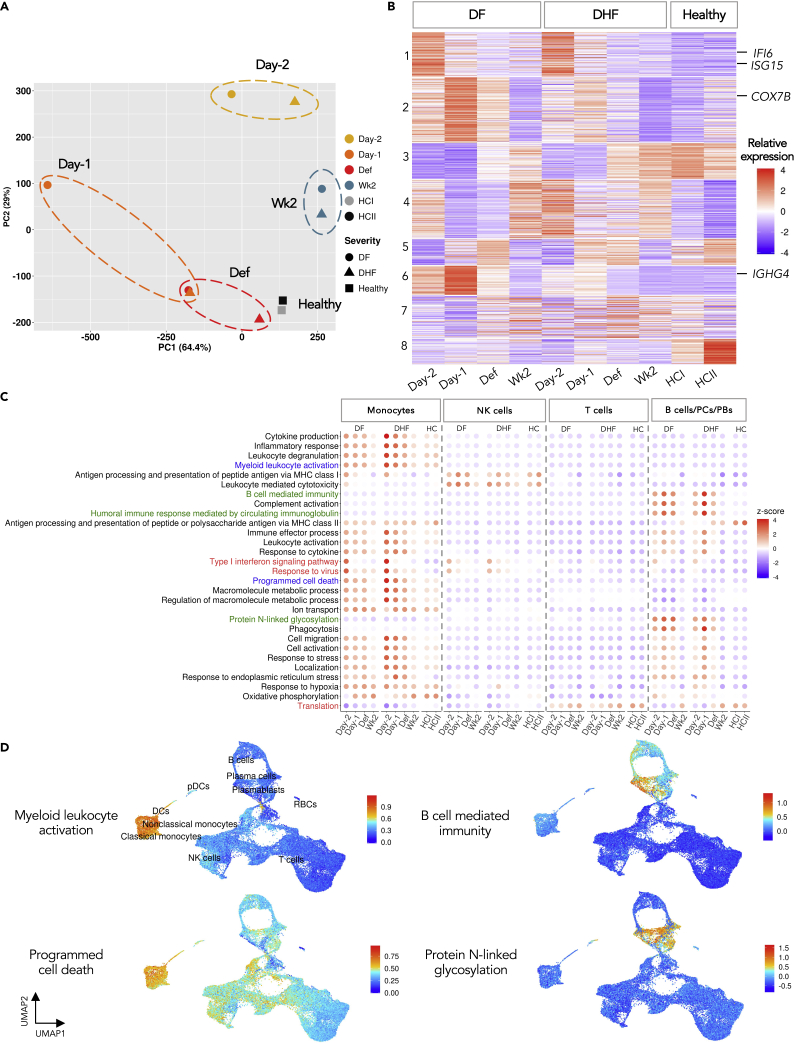
Time-course transcriptomic profiling reveals detailed molecular events in the febrile phase of DENV infection (A) Principal Component Analysis (PCA) of overall transcriptomic patterns among the ten PBMC samples. Colors represent the time points of DENV infection; shapes represent different severities. (B) Heatmap showing the relative expression of highly variable genes (HVGs), from the union of the top 500 genes in PC1 and PC2 from (A) (see Method details). (C) Dotplots illustrating the relative expression of HVGs over the course of DENV infection. The biological processes (BPs) of HVGs that are common across the four cell types are in red; monocyte-specific BPs are in blue, and B cell-specific BPs are in green. The relative expression is indicated by the color intensity (see Method details). (D) UMAP feature plots of the relative signature scores of HVGs that are associated in that particular BPs (see also Figure S4).

We next visualized these average transcriptomic profiles of the ten samples using Principal Component Analysis (PCA) (Figure 2A). The two independent HCs, HCI from this study and HCII from a public dataset (see STAR Methods), are situated nearly on top of each other, suggesting that their overall transcriptomic profiles were very similar. We observed that the Wk2 samples of both patients were grouped together, and appeared to be in close proximity to HCs along the first principal component (PC1), which accounts for over 64% for the transcriptional variations. Together, these suggest that to a large extent the Wk2 samples could be considered as the baseline transcriptional profiles for both patients. Well in line with the overall correlation coefficients (Figure S2), the PCA confirmed that the average transcriptomic profiles of Day −1 were the furthest from Wk2 in both patients. Interestingly, the largest different profiles between the patients of different severities at the matched time points (Figures 2A and S2) were also seen at Day −1.

We then extracted “Highly Variable Genes” (HVGs), which represent the genes that demonstrated the largest changes in expression across the ten samples (Table S3, see also Method details). Their pseudo-bulk transcriptomic patterns already showed several unique transcriptomic changes across the time points and between the patients and controls (Figure 2B). Among the enriched biological processes (BPs) of the HVG clusters, type I interferon responses were up-regulated in both patients as early as Day −2 (Figure 2B, Cluster 1; *e.g., IFI6* and *ISG15*). The expression peaks of the oxidative phosphorylation HVGs (Cluster 2, *e.g., COX7B*) and B cell receptor signaling pathway HVGs (Cluster 6, *e.g., IGHG4*) were observed at Day −1 (Figure 2B). These results suggest that the DENV-infected transcriptomes were temporally specific and already observable even at the level of population-wide transcriptomes. The complete list of genes in transcriptionally unique clusters and their associated GO terms can be found in Tables S4 and S5.

### Transcriptional dynamics of cell-type specific immune pathways in DENV infection

We next explored the cell-type specific changes in the transcriptional dynamics across the course of infection by re-extracting the HVGs of four major immune cell lineages: monocytes, NK cells, T cells and B cells/PCs/PBs (Figures 2C and S3, see full lists of HVGs in Tables S6, S7, S8, and S9 and the percentages of cells expressing HVGs in Table S10). To investigate the expression dynamics of the cell-type HVGs and their enriched BPs (Table S11), relative transcriptional changes across the ten samples and the four cell types were computed (Figure 2C; Tables S12, S13, S14, and S15, see also Method details).

There are three BPs whose gene members are HVGs in all four cell types, namely “type I interferon signaling pathway” (*e.g., ISG15*, *IFI6*, and *GBP1*), “responses to virus” (*e.g., EIF2AK2*, *RUNX3*, and *PARP9*) and “translation” (*e.g., RPL22*, *RPL15*, and *RPS8*) (Figure 2C, highlighted in red). Several genes related to type I interferon and responses to viral infection were relatively up-regulated at Day −2 in both patients, and then declined as the disease progressed (Figures 2C and S4). This pattern was observed in each of the four populations, however, it was most prominent in monocytes (Figures 2C and S4). Type I interferon responses are known to be a crucial protective immune-mechanism against viral infection (Akira, 2009; Diamond et al., 2000). Indeed, the up-regulation of type I interferon responses at Day −2 and declining toward Def, corresponded to the high viral loads at febrile days, before becoming undetectable at defervescence in both patients (Table S16), as also observed in previous studies (Ben-Shachar et al., 2016; Matangkasombut et al., 2020). These together suggest that type I interferon responses are one of the early antiviral mechanisms generally elicited by immune cells against the DENV infection. By contrast, the “translation” HVGs, consisting primarily of ribosomal and translation initiation/elongation genes, were down-regulated during febrile DENV infection in all four cell types (Figures 2C and S4). Interestingly, it has been shown *in vitro* that the infections of DENV and Zika virus, can repress the host cell translation while maintaining their own protein synthesis (Roth et al., 2017). Our result suggests that this event might occur specifically during the febrile stage of natural DENV infection in all four immune cell types investigated.

We next looked at the cell-type specific BPs (whose genes are HVGs only in particular cell types). In B and antibody-secreting cells (ASCs, including PBs and PCs), as expected we observed HVGs that are functionally enriched in “B cell mediated immunity” (*e.g., FCER1G*, *CD74*, *CD70*), “humoral immune response mediated by circulating immunoglobulin” (*e.g*., *IGHA1*, *IGLC3*, *IGHM*) and also “protein N-linked glycosylation” (*e.g., DDOST*, *DAD1, OST4*). These HVGs were relatively up-regulated during Day −1 in both DF and DHF (Figure 2C, highlighted in green, also Figures 2D and S4). Hence, in addition to the expansion of the ASC populations (Figure 1C), their antibody production activities also appeared to be enhanced at Day −1. The up-regulation of the N-linked glycosylation process, in conjunction with immunoglobulin production of ASCs in the febrile phase, suggests that post-translational modification might also play a role in the antibody function in response to DENV infection. Indeed, the effect of antibody N-linked glycosylation pattern on the binding affinity of Fc receptors has been demonstrated in viral infections (Irvine and Alter, 2020). However, further study is still needed to explore the clonality and antigen specificity of the antibody produced, and if the N-glycosylation activity observed here indeed modifies the IgG Fc portion.

The BPs of the HVGs specifically activated in monocytes at Day −2 include “myeloid leukocyte activation” (*e.g.,* *C5AR1* and *FCER1G*) and “programmed cell death” (*e.g., BCL2A1*, and *TNF*) (Figure 2C, highlighted in blue, also Figures 2D and S4). The activation of signature genes of monocytes and other myeloid cells is among the indicators of early immune responses to DENV infection (Kwissa et al., 2014). Because monocytes are one of the primary targets of the infection, they serve as the source of proinflammatory cytokines that contribute to the pathogenesis of DENV infection (Castillo and Urcuqui-Inchima, 2018). Notable abundant genes that also showed highly dynamic expression across the infection course in monocytes include *S100A8* and *S100A9*, which were also up-regulated at Day −2 (Figure S5). The two genes encode calprotectin, an antimicrobial protein family, which are associated with multiple BPs, including “cytokine production”, “inflammatory responses”, and “leukocyte degranulation”. These proinflammatory proteins, also shown to be highly expressed in the monocytes of severe COVID-19 patients, are potent stimuli of neutrophils (Xu et al., 2020). In DENV, the potential pathogenic role of neutrophils against the infection has already been shown (Opasawatchai et al., 2018), suggesting a possible connection between the high expression of *S100A8/9* at Day −2 and DENV pathogenesis.

### Functional characterization of T cell subpopulations in DENV infection

**Figure 3:**
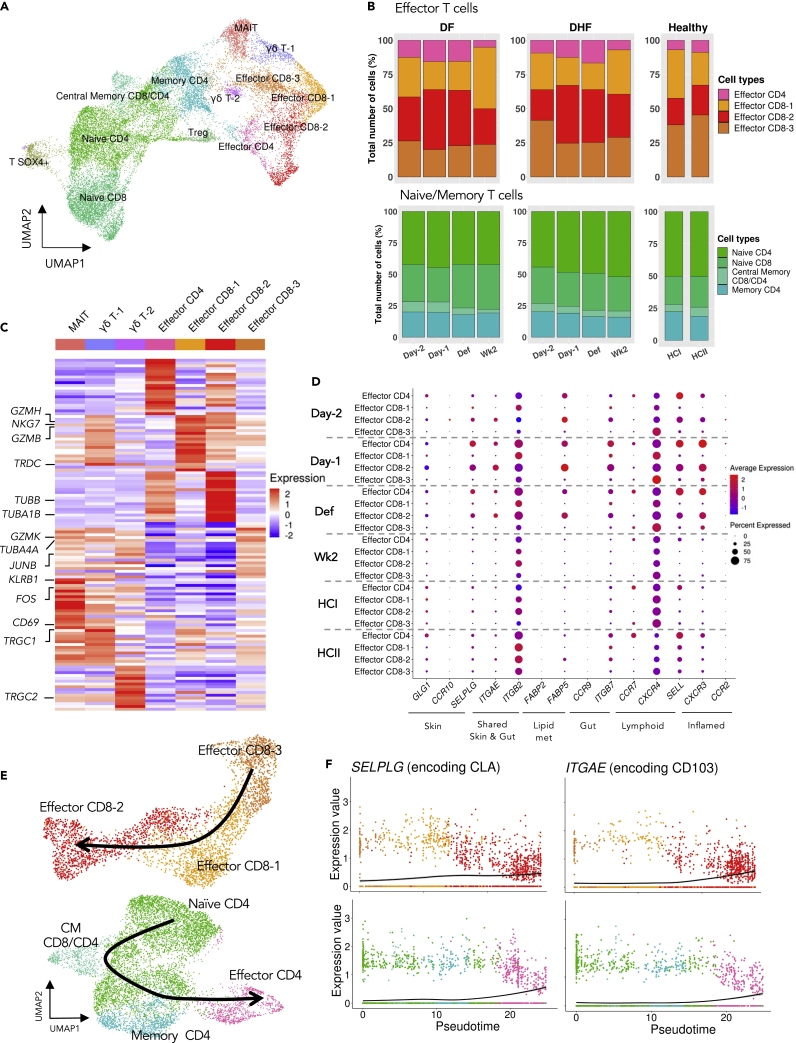
Functional characterisation of T cell subpopulations during DENV infection (A) UMAP plots of integrated T cell transcriptome profiles from the ten PBMC samples (see also Figure S6 and Table S1). (B) Relative abundances of Effector T cells (upper panel) and Naive/Memory T cells (lower panel). (C) Relative expression of the top 20 differentially expressed genes (DEGs) between each of the subpopulations and the rest of effector-like T cells. (D) Average expression of tissue-homing genes in Effector T cells. The dot sizes represent the proportion of cells expressing the genes. (E) UMAP plots showing the Effector CD8 (upper panel) and CD4 (lower panel) T cells. Black lines represent pseudotime constructed by Monocle3 (Cao et al., 2019), by setting Effector CD8-3 and Naive CD4 as roots (see also Figure S13). (F) Pseudotime kinetics of *SELPLG*, and *ITGAE* from the roots in Effector CD8 (upper panel) and CD4 (lower panel) T cells (see also Figures S13–S15). The color codes for T cell subpopulations are as in (E).

The involvement of heterogeneous T cell populations in DENV infection is complex in several aspects, notably the dynamic changes during the infection period (Dung et al., 2010) and their roles in both protective (Weiskopf et al., 2013; Yauch et al., 2009) and pathogenic responses (Duangchinda et al., 2010; Mongkolsapaya et al., 2003). This is likely because of highly functionally and phenotypically heterogeneous subpopulations of T cells, as well as the limited time points focused in previous studies (Screaton et al., 2015; Tian et al., 2019). Based on the distinct transcriptional patterns, we further characterized T cell subpopulations, including the Naive/Memory-like, Effector, MAIT, Gamma Delta (γδ), and regulatory (Treg) T cells (see Figures 3A and S6; Table S1 for expression of T cell molecular markers).

Among the effector T cell subpopulations, we identified three transcriptionally distinct groups of CD8^+^ effector T cells (referred to as “Effector CD8-1, −2 and −3” herein), and a group of “Effector CD4” T cells (Figures 3A and S6; Table S1). Interestingly, the relative abundances of effector T cell subpopulations appeared to be variable across the infection period, with the Effector CD4 and Effector CD8-2 expanding the most around Day −1 and Def in both patients (Figure 3B, pink and red). On the contrary, the relative abundances of the Naive/Memory-like subpopulations (Naive CD4 and CD8, Central memory CD8/CD4, and Memory CD4) were largely unaltered throughout the time points, and also present at similar percentages to those of HCs (Figure 3B, green and blue).

Looking further into the effector T cell subgroups, we observed highly transcribed genes, including the NK-like and cytotoxic features such as *NKG7, GZMB* and *GZMH* in Effector CD8-1. In Effector CD8-2, we found high expressions of *GZMB* together with cell adhesion and proliferation molecules such as *TUBB* and *MKI67* and also moderate expression of the exhaustion marker *PDCD1* (Figures 3C and S7–S10). Effector CD8-3 highly expressed *GZMK* and genes associated with inflammation and leukocyte activation such as *JUNB*, *FOS,* and *CD69.* For Effector CD4, we also observed the cytotoxic markers *GZMA* and *GZMK*, adhesion gene *TUBB* (Figures 3C and S10)*.*

Several innate-like T cells have been shown to participate in early rapid response to DENV and other viral infections, including γδ T cells (Caron et al., 2021; Mantri and St John, 2019; Tsai et al., 2015), MAIT (Paquin-Proulx et al., 2018; van Wilgenburg et al., 2016) and invariant NKT (iNKT) cells (Matangkasombut et al., 2014; St John et al., 2011). For the γδ T cells, we observed two subclusters with distinct transcriptomic patterns (Figures 3C and S11), the larger population expressing the markers *TRDC*, *TRGC1*, *TRGC2*, *KLRB1* (which encodes the CD161 protein), and the other expressing *TRDC*, *TRGC2*, but not *KLRB1*. We termed them γδ T Groups 1 (γδ T-1) and 2 (γδ T-2), respectively (Figure S6 and Table S17). CD161, a c-type lectin-like receptor, is expressed in several T cell populations associated with IL-17 or TNF/IFNg production (Truong et al., 2019), falling in line with the functional link between γδ T and Th17 cells. During the febrile phase, γδ T-1 cells expressed genes that are associated with lymphocyte activation (*CD69*), cytotoxicity (*GZMB* and *GZMK*) and inflammation (*IL32* and *NFKBIA*) (Figures 3C and S11), suggesting its possible role in defense against DENV.

### Effector CD4 and effector CD8-2 T cells expressed tissue-homing signature genes during febrile period

Effector CD4 T cells and Effector CD8-2 displayed some similar transcriptional patterns, suggesting they might be involved in overlapping molecular pathways and perform similar effector functions patterns in response to DENV infection (Figures 3C and S9). It has been shown that the expression of adhesion molecules such as *TUBB*, *TUBA1B* and *LGALS3,* together with a cell proliferation marker *MKI67,* are associated with tissue-derived T cells (Szabo et al., 2019). Hence, we speculated that Effector CD8-2 and Effector CD4 might possess a signature tissue-homing molecule associated with the potential sites that these T cells might home to. Indeed, unlike other effector subgroups, Effector CD8-2 and Effector CD4 expressed specific skin-homing signature genes such as *GLG1* (encoding an E-selectin ligand) and *SELPLG* (encoding the CLA protein), as well as the inflammatory signature *CXCR3* during the febrile period (Day −1 and Def) in both patients (Figures 3D and S12). This suggests that the two effector T cells might respond to natural DENV infection by homing to skin, where the virus enters the body, and falls in line with a previous study showing that CLA and CXCR3 were expressed in DENV-specific CD4^+^ and CD8^+^ T cells (Rivino et al., 2015). Of interest, we also found that *FABP5*, a lipid binding molecule associated with skin residency (Frizzell et al., 2020), was expressed in Effector CD4 and Effector CD8-2 (Figure 3D). In addition, the two effector T cells also expressed other tissue-homing genes such as *ITGAE* (skin and gut), and *ITGB7* (gut).

We next computed the “cellular trajectory” or “pseudotime” to investigate the transcriptional states and functional lineages of the T cell subpopulations. Focusing on the Effector T cells, Effector CD8-2, which highly transcribed a number of tissue-homing genes, appeared in the more advanced state of the predicted pseudotime (Figures 3E, S13, and S14). Well in line with this, we observed that key tissue-homing genes, including *SELPLG1* and *ITGAE,* were up-regulated along the pseudotime axis, especially in Effector CD8-2 (Figure 3F). For the CD4^+^ T cells, Effector CD4 T cells were also placed toward the end of the trajectory, and the two tissue-homing genes were more highly transcribed in the advanced state of the CD4 T cell pseudotime (Figures 3E, S13, and S15), providing further evidence about the role of effector T cells in responding against the DENV-infection by mobilizing toward the skin, the initial organ through which the pathogen enters the host.

### Expansion of the skin-homing CD69^+^ PD-1^+^ T cell clusters was shown in additional samples using flow cytometry

**Figure 4:**
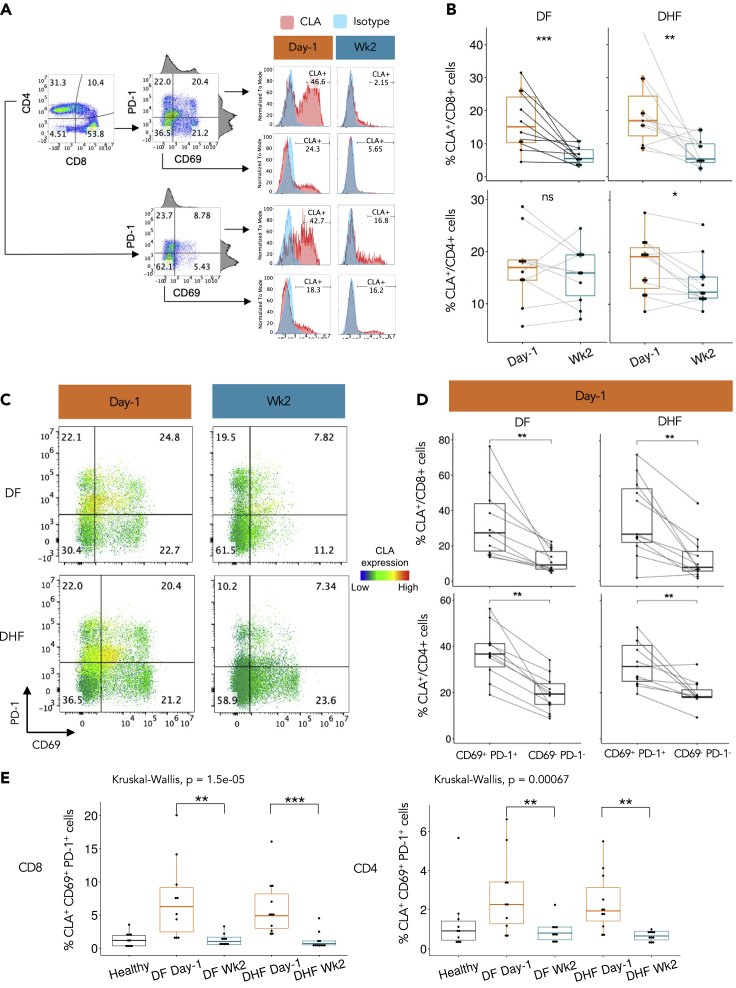
Flow cytometry analyses confirmed upregulation of the skin-homing marker CLA in the CD69^+^ PD-1^+^ T cells at one day before defervescence (A) Gating strategy of the CD4^+^ and CD8^+^ T cells (left panel). Histograms (right panel) showing the gating of the CLA^+^ cells, with the numbers representing the percentages of the CLA^+^ cells. The CLA stained samples are in red and isotype controls are in blue. (B) Percentages of the CLA^+^ cells in the CD8^+^ (upper lanel) and CD4^+^ (lower panel) T cell populations at Day −1 and Wk2 of the same patients. (C) Relative expression levels of CLA in the CD69^−^ PD-1^+^, CD69^+^ PD-1^+^, CD69^+^ PD-1^-^, and CD69^−^ PD-1^-^ populations of the CD8^+^ T cells. The number in each quadrant represents the percentage of cells (see Figure S17 for CD4^+^ T cells). (D) Percentages of the CLA^+^ cells in the CD69^+^ PD-1^+^, as compared to CD69^−^ PD-1^-^, in the CD8^+^ (upper panel) and CD4^+^ (lower panel) T cells in 10 DF (left panel) and 11 DHF (right panel) patients at Day −1. (E) Percentages of the CLA^+^ CD69^+^ PD-1^+^ cells in the CD8^+^ (left panel) and CD4^+^ (right panel) T cell subpopulations. Wilcoxon signed-rank test was used to analyze the differences in percentages of the CLA^+^ cells between two given time points of the same patients. Kruskal-Wallis test followed by Dunn’s test with a Benjamini-Hochberg method, was used to analyze the differences in percentages of the CLA^+^ cells in the T cell subpopulations among multiple samples. ns = p > 0.05, ∗p ≤ 0.05, ∗∗p ≤ 0.01, ∗∗∗p ≤ 0.001, and ∗∗∗∗p ≤ 0.0001. Healthy controls, n = 10; DF patients, n = 10; DHF patients, n = 11.

To further investigate the potential roles of the Effector CD8-2 and Effector CD4 T cells in response to natural DENV infection, we analyzed the expression of a skin-homing signature molecule, CLA (encoded by *SELPLG*), using flow cytometry (Figure 4A). Based on the samples from 10 additional DF and 11 DHF patients during the febrile illness (Day −1) and matched 2-week convalescence (Wk2), there was no difference in the proportions of CLA-expressing cells in the whole CD4^+^ and CD8^+^ T cell populations between either group of DENV patients, as compared to the 10 HCs (Figure S16). We did observe that CLA was expressed higher in the CD8^+^ T cells at Day −1 in both DF (pvalue ≤ 0.001, Wilcoxon signed-rank test) and DHF (pvalue ≤ 0.01) patients, than those at Wk2 (Figure 4B), but not in the CD4^+^ T cells.

In addition to *SELPLG*, our scRNA-seq data also suggested differential expression of the activation marker (*CD69*) and Programmed cell death protein 1 (*PDCD1*) among the three Effector CD8 subgroups. While Effector CD8-1 transcribed relatively low levels of both *PDCD1* and *CD69*, Effector CD8-3 transcribed high levels of *CD69* but low *PDCD1* (Figures S7 and S8). Interestingly, both Effector CD8-2 and Effector CD4 showed high transcription levels of *PDCD1* and moderate levels of *CD69*, especially during acute infection. Hence, we went on to investigate whether the CLA protein might be up-regulated in the CD69^+^ PD-1^+^ cells, as seen at the transcriptional level. Indeed, the CLA expression level was highest in the CD69^+^ PD-1^+^ populations in both the CD8^+^ and CD4^+^ T cells at Day −1 (Figures 4C, 4D and S17). Intriguingly, when we looked into the CLA^+^ CD69^+^ PD-1^+^ cells in the CD4^+^ and CD8^+^ T cell populations, the proportions of the cells expressing these three surface markers were significantly higher at Day −1 in both DF (pvalue ≤ 0.01) and DHF (pvalue ≤ 0.001) (Figure 4E, left), and the same were seen in the CD4^+^ T cells (pvalues ≤ 0.01) (Figure 4E, right). Taken together, we have validated the expression of the skin-homing signature protein CLA during febrile DENV infection in specific groups of effector CD8^+^ and CD4^+^ T cells expressing CD69 and PD-1 (as seen in Effector CD8-2 and Effector CD4 at the transcription level). This is not the case, however, for CD103 (encoded by the *ITGAE* gene) another homing marker described earlier, possibly because the protein could be detected only at very low levels (Figure S18).

### Expansion of plasma cells and plasmablasts and their tissue-homing signatures during febrile phase

**Figure 5:**
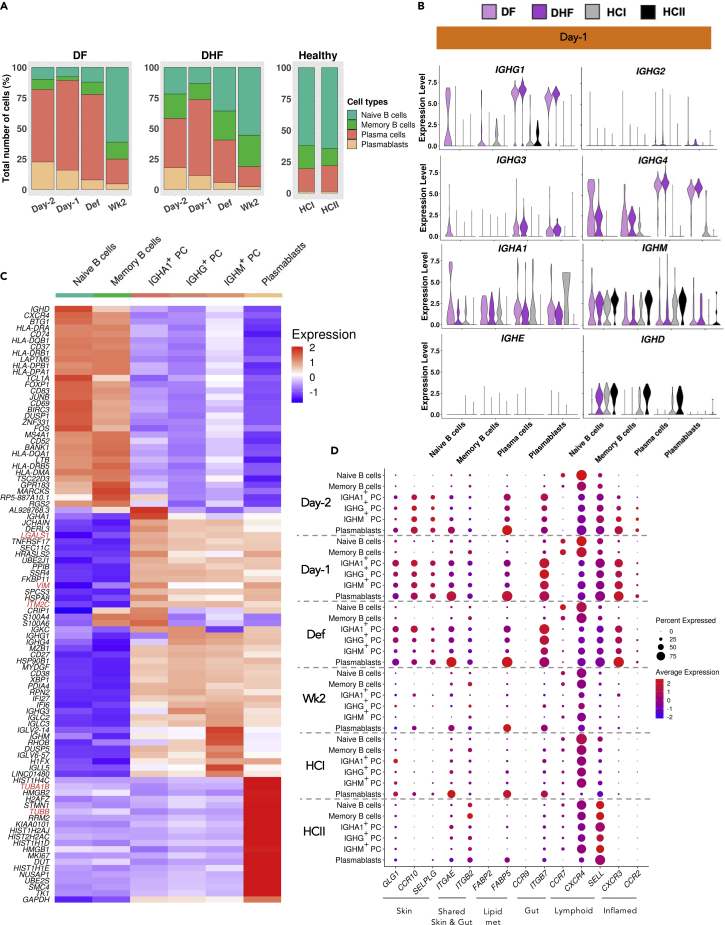
Functional characterisation of B cells, PCs, and PBs during DENV infection (A) Relative abundances of B cell subpopulation in each sample (see also Table S2). (B) Violin plots representing the normalized transcription levels of the major IG genes at Day −1 in each B cell subpopulation (see also Figure S20). (C) Heatmap representing the relative expression of the top 20 differentially expressed genes between each of the subpopulations and the rest of B cells. (D) Average expression of tissue-homing genes in each B cell subpopulation across time of DENV infection. The dot sizes represent the proportions of cells expressing the genes.

PCs, PBs, and B cells, are the main producers of antibodies, which in turn can play both protective and pathogenic roles in responses to DENV (Rey et al., 2018). Here, we observed the relative expansion of PC and PB populations in acute DENV infection at Day −1 in both DF and DHF patients, as compared to at Wk2, and also the two HCs (Figures 1C and 5A).

To further investigate the gene expression characteristics and putative functions of B cells and related ASCs (*i.e.,* PBs and PCs) during the course of DENV infection, we looked into the distributions of immunoglobulin (Ig) subclasses which define the binding to particular Fc receptors and antibody-dependent cell-mediated cytotoxicity. Of interest, the main Ig genes specifically transcribed during the acute infection were *IGHG1* and *IGHG4* in ASCs of both DF and DHF patients, as compared to at Wk2 or in HCs (Figures 5B, S19, and S20). Looking at the genes specifically transcribed in different B cell-related subpopulations, we observed several cycling and tissue-homing signatures, namely *MKI67*, *TUBB*, *VIM* and *LGALS1* in PBs (Figure 5C), similarly to in the Effector CD4 and Effector CD8-2 T cells described earlier. We then further explored transcription of tissue-homing signature genes, and found several skin-homing (*GLG1*, *CCR10,* and *SELPLG*), gut-homing (*ITGB7*, *ITGAE*) and inflamed tissue-homing (*CXCR3* and *CCR2*) signatures in ASCs specifically during acute infection in both patients, but not at Wk2 nor in HCs (Figures 5D and S21). The up-regulation of inflamed tissue-homing proteins, namely *CXCR3* and *CCR2*, in ASCs have already been reported in DENV-infected patients (Pattanapanyasat et al., 2018), but not the skin- and gut-homing markers. Taken together, our results suggested that the unique subpopulations of T and B lymphocytes expressed tissue-homing signatures specifically during the febrile period (Figures 3D, 5D, S7–S9, S12, S14, S15, and S21), and might play a role in localized immune response against the DENV infection.

## Discussion

The clinical manifestation of symptomatic DENV infection progresses rapidly during the febrile phase, followed by a very narrow window of critical period, when hemorrhagic shock and death could occur without timely and appropriate patient management (World Health Organization, 2009). Unfortunately, there is still no definitive early diagnostic biomarker of disease progression that would ensure accurate triage and effective patient management (Kalayanarooj, 2011; Muller et al., 2017). Furthermore, better insights into the immune responses during the febrile phase is still required to improve early intervention that could prevent adverse disease outcomes (St John and Rathore, 2019; Tsai et al., 2017).

Our study provides a comprehensive analysis of detailed time-course scRNA-seq of the immune responses during acute natural DENV infection. The initial explorative characterisations of the immune cell subpopulations with distinct expression dynamics and cellular functions were based on two dengue patients, one with DF and the other with DHF, with matched gender (male), serotype (DENV-4). We focused on the secondary infection because it is associated with more severe symptoms as compared to the primary infection (Guzman et al., 2016; World Health Organization, 2009). The detailed cell-type identifications and gene expression analyses have been performed across four different time points, which were carefully determined in relation to defervescence, one of the most critical milestones of the infection course. With three time points during the acute period and one at two-week follow-ups, to the best of our knowledge, this study provides the most detailed temporal single-cell transcriptomic analysis in the patients with natural DENV infection so far.

With the detailed analyses of temporal scRNA-seq profiles, we observed intriguingly consistent trends between the two DENV patients throughout the four time points, both in terms of the dynamics of relative abundances of immune cell types, as well as overall and cell-type specific gene expression patterns. These allowed us to improve the depth of earlier transcriptomic analyses of the host immune response against DENV, which might partly be restricted by the low-resolution bulk RNA-seq and microarray (Hanley et al., 2021; Popper et al., 2012; Sun et al., 2013; van de Weg et al., 2015). Although most of the time-course RNA-seq studies of immune response in dengue patients or *in vitro* systems were performed using the samples collected during the vaguely defined “acute infection” or “febrile phase” (Hanley et al., 2021; Sun et al., 2013; van de Weg et al., 2015; Waickman et al., 2021), we broke this important period down to three consecutive days, analyzed the single-cell transcriptomic profiles of DENV patients for each day, and compared those with the two-week follow-up baselines.

Through cell-type identifications using established molecular markers, we were able to simultaneously monitor the relative abundances of key immune cell populations of the two patients through the course of infection, as compared to those of the two healthy controls. Despite the different sample preparation protocols and most likely different ethnic groups, the two HCs showed intriguingly similar distributions of immune cell types in the PBMC populations (Figure 1C) and gene expression patterns (Figures 2A–2C), and were also closely related to those of the patients at Wk2. In both patients, we observed relative expansion of T cells at Def, and that of PCs and PBs at Day −1. While both PCs and PBs are normally scarce in PBMCs of healthy donors, plasmacytosis could be seen in several pathologic conditions (Thai et al., 2011; Wrammert et al., 2012), well in line with the expansion and PCs and PBs previously observed during the febrile phase of DENV infection (Garcia-Bates et al., 2013; Kwissa et al., 2014).

Here, we focused on the dynamic expression and functional characteristics of the immune cells in the PBMC samples and their subpopulations. We have demonstrated the expression peaks of several highly variable gene (HVG) modules that appeared to vary greatly even within a short time frame of the febrile period (Figures 2B and 2C). At Day −2, the earliest time point in our study, we already observed the up-regulation of genes associated with type I interferon and response to virus in all major immune cell types (Figure 2C). This is in line with previous reports showing prominent expression of type I interferons at early phases of DENV infection (Popper et al., 2012; Sun et al., 2013; van de Weg et al., 2015; Waickman et al., 2021), even though the exact timelines of the infection were characterized slightly differently in each study. In this study, we were able to pinpoint that the type I interferon pathway was most pronounced in monocytes. In earlier studies, the down-regulation of ribosomal and translation genes were seen in early DENV infection (Hanley et al., 2021; Roth et al., 2017; Waickman et al., 2021). Our detailed analysis showed that the translation-related genes were suppressed the most at Day −2 in monocytes and B cells, which are known to be the direct targets of DENV (Durbin et al., 2008; Upasani et al., 2020; Zanini et al., 2018), and to a lesser extent in non-DENV-targeted populations such as T cells.

One day before the fever subsided (Day −1) marked a critical molecular checkpoint of the dengue progression, where several key events occurred, including the relative expansion of B and ASCs (Figures 1C and 5A). More specifically on the functions of these ASCs during acute infection, we also discovered the up-regulation of particular Ig genes in PBs and PCs at Day −1 (Figure 5B), and several tissue-resident and skin-homing genes in PBs (Figures 5C and 5D). Using scRNA-seq, we demonstrated transcriptional upregulation of other genes associated with tissue resident lymphocytes, including *GLG1* and *SELPLG* in PBs and PCs of the DENV patients during acute DENV infection (Figure 5D). Of interest, we observed that several predictive genes of severe dengue, including *GYG1, TOR3A, SPON2, GRAP2 and GBP2* (Robinson et al., 2019), were also highly transcribed in the ASCs and effector T cells at Day −1 in our dataset (Figures S22–S24). These together suggest that the expression levels of key genes at one day before defervescence are important not only in terms of disease progression, but potentially also severity prediction. We noted, however, that our study was not specifically designed to investigate the differences between the severities, and future studies on larger groups of patients with different severities would be needed to validate this observation.

Unlike B cells, T cells are not known as a direct target of DENV, but their functions during the DENV infection are known to be complex and important for mediation of the immune responses against the virus in several aspects (Screaton et al., 2015; Tian et al., 2019; Weiskopf et al., 2013). In addition to the major immune cells that can be identified by well-established markers, scRNA-seq also allowed us to explore and characterise their subpopulations with unique dynamic expression and possibly meaningful cellular functions against DENV infection. Also at Day −1, we observed the expansion of tissue-homing T cell subpopulations, Effector CD4 and Effector CD8-2, which were initially characterized and explored using the scRNA-seq profiles from two DENV patients (one DF and one DHF, Figures 3C and 3D), and further validated using additional 10 DF and 11 DHF patients by the flow cytometry (Figure 4).

The two effector T cell subpopulations, Effector CD8-2 and Effector CD4, demonstrated the highest transcription levels of the marker genes of proliferation (*MKI67*), cytotoxicity (*GZMA* and *GZMK* in CD4 and *GZMB* in CD8-2), inflammation (*CXCR3*), skin and gut homing (*SELPLG* and *ITGB7,* respectively) (Figures 3C, 3D, and S7–10) at Day −1, suggesting that they might be activated and eventually home to the infected tissues in response to DENV infection. Several studies have highlighted important roles of tissue-resident memory T (Trm) cells in the immune responses against viral infections at the barrier surfaces in vaccinia virus (Jiang et al., 2012; Schenkel et al., 2014), lymphocytic choriomeningitis virus (Kurd et al., 2020; Schenkel et al., 2014), and SARS-CoV-2 (Grau-Exposito et al., 2021). For DENV, Rivino et al. have shown that the circulating DENV-specific CLA^+^ CD8^+^T cells were expanded during the acute phase and DENV-specific T cells were found in the skin of the patients (Rivino et al., 2015). Based on the lipid binding molecules, inflammation and skin homing markers, and other genes that were shown to be associated with Trm (Frizzell et al., 2020; Szabo et al., 2019), we speculated that Effector CD8-2 and CD4 T cells might serve as the precursor of, or at least be associated with DENV-specific Trm, which rapidly home to skin, the initial infected tissue of DENV through mosquito bites, right before the critical period. The HLA-DR^+^ CD38^+^ CD8^+^ T cells have been shown to expand and express marker genes for proliferation, tissue homing, and cytotoxic functions; as well as become unresponsive to IFN-gamma and develop TCR refractoriness in dengue patients (Chandele et al., 2016). Interestingly, we also observed up-regulation of overlapping makers of cell proliferation (*e.g.,* *MKI67, TOP2A*), tissue homing (*e.g.,* *SELPLG*) and co-inhibitory molecules *(e.g.,* *CTLA4, LAG3*) in our CD8-2 T cells during the acute phase (Day −2 and Day −1) in both DF and DHF (Figure S25), suggesting that these effector T cells might be related to the HLA-DR^+^ CD38^+^ CD8^+^ T cells described by Chandele et al..

In addition to the tissue-homing markers, we also observed moderate transcription of the cell death marker *PDCD1* gene in Effector CD8-2 and CD4 T cells (Figure S8). According to Alwis et al., a population of proliferative DENV-specific CD8^+^ T cells that displayed effector-memory phenotype, were functionally active during acute DENV infection, despite also expressing the cell-death maker PD-1 (de Alwis et al., 2016). In COVID-19 patients, a PD-1^+^ subpopulation of SARS-CoV-2-specific CD8^+^ T cells has also been shown to be functional rather than exhausted, suggesting that the PD-1 upregulation could be observed as a result of early T cell activation (Rha et al., 2021). All in all, this study provides an unprecedented in-depth understanding of the detailed dynamic immune response during natural DENV infection and lays the foundation for the development of predictors of disease progression for better patient triage, and to improve clinical management of DENV patients.

### Limitations of the study

We noted, however, that the study is still restricted by the technical limitations to investigate the clonality of T and B cells in the DENV-infected patients, which can now be addressed using more recently developed scRNA-seq methods that can analyze the TCR and BCR sequences (Pai and Satpathy, 2021; Singh et al., 2019); and to look into the DENV-specific immune cells, which can now be investigated using surface protein expression (Chng et al., 2019; Stoeckius et al., 2017) and DENV-epitope loaded HLA tetramer technology. Because the transcription and translation levels do not always correlate, candidate genes from differential expression analyses can also be followed up by single-cell mass cytometry (Bendall et al., 2011) and flow cytometry. A substantially large group of patients will be essential to elucidate the factors that contribute to different dengue severities, or the protective and pathogenic functions of different immune cell types. Single-cell transcriptomic analyses of such large cohorts have so far been hindered by the funding limits, but now can be achieved in a much more cost-effective manner (Kang et al., 2018; Stoeckius et al., 2018).

## Consortia

DENFREE Thailand : Anavaj Sakuntabhai (Functional Genetics of Infectious Diseases Unit, Institut Pasteur, Paris, France, Centre National de la Recherche Scientifique (CNRS), URM2000, Paris, France), Pratap Singhasivanon (Department of Tropical Hygiene, Faculty of Tropical Medicine, Mahidol University, Bangkok, Thailand), Swangjit Suraamornkul (Endocrinology Division, Department of Medicine, Faculty of Medicine Vajira Hospital, Navamindradhiraj University, Bangkok, Thailand), Tawatchai Yingtaweesak (Thasongyang Hospital, Tak, Thailand), Khajohnpong Manopwisedjaroen (Department of Microbiology, Faculty of Science, and Faculty of Tropical Medicine, Mahidol University, Bangkok, Thailand), Nada Pitabut (Faculty of Tropical Medicine, Mahidol University, and Faculty of Medicine, King Mongkut’s Institute of Technology Ladkrabang, Bangkok, Thailand).

## STAR★Methods

### Key resources table

**Table:** 

REAGENT or RESOURCE	SOURCE	IDENTIFIER
**Antibodies**		

PerCP anti-human CD3 Antibody	Biolegend	Cat#300326; RRID: AB_2616610
Brilliant Violet 510™ anti-human CD4 Antibody	Biolegend	Cat#357420; RRID: AB_2715940
APC/Cyanine7 anti-human CD8 Antibody	Biolegend	Cat#344714; RRID: AB_2044006
Brilliant Violet 421™ anti-human CD69 Antibody	Biolegend	Cat#310930; RRID: AB_2561909
Alexa Fluor® 700 anti-human CD279 (PD-1) Antibody	Biolegend	Cat#329952; RRID: AB_2566364
APC anti-human CD103 (Integrin αE) Antibody	Biolegend	Cat#350216; RRID: AB_2563907
PE anti-human/mouse Cutaneous Lymphocyte Antigen (CLA) Antibody	Biolegend	Cat#321312; RRID: AB_2565589
Brilliant Violet 421™ Mouse IgG2a, κ Isotype Ctrl Antibody	Biolegend	Cat#400260
Alexa Fluor® 700 Mouse IgG1, κ Isotype Control	BD Bioscience	Cat#557882, RRID: AB_396920

**Biological samples**		

Human PBMC samples	The Institutional Review Boards of Faculty of Medicine Vajira Hospital (No.015/12), Faculty of Tropical Medicine Mahidol University (TMEC 13041) and Faculty of Medicine, Ramathibodi Hospital, Mahidol University (MURA2019/603).	N/A

**Chemicals, peptides, and recombinant proteins**		

Fetal bovine Serum (FBS)	Invitrogen	10270
RPMI 1640 Medium	Gibco™	Cat# 11875085
IsoPrep Isolation Medium for Separation of Human Lymphocytes	Robbins Scientific Corporation	1070-04-0
Bovine Serum Albumin (BSA)	Sigma-Aldrich	A7030-100G
Dimethyl sulfoxide (DMSO)	Sigma-Aldrich	D2650-5X5ML
Dulbecco′s Phosphate Buffered Saline (DPBS)	Gibco™	14190-144
Paraformaldehyde	Sigma-Aldrich	Cat# 30525894

**Critical commercial assays**		

Death cell removal kit	Miltenyi Biotec	Cat# 130-090-101
Agilent High Sensitivity DNA D1000 Screen tape	Agilent	Cat# 5067-5584
Agilent High Sensitivity DNA D1000 reagents	Agilent	Cat# 5067-5585
Agilent High Sensitivity DNA D1000 ladder	Agilent	Cat# 5067-5587
Qubit™ dsDNA HS Assay Kit	Thermo Fisher Scientific	Cat# Q32851
Chromium Single Cell 3′ Library Kit v2, 16 rxns	10x Genomics	Cat# 120234
Chromium Single Cell 3′ Gel Bead Kit v2, 16 rxns	10x Genomics	Cat# 120235

**Deposited data**		

Dataset: Raw sequencing data of 4 time-points from DF and DHF patients and a healthy donor	This study	ArrayExpress : E-MTAB-9467
Dataset: 4k PBMCs from a healthy donor	10x Genomics Single Cell Gene Expression Datasets	https://support.10xgenomics.com/single-cell-gene-expression/datasets/2.1.0/pbmc4k
Algorithms and computer codes	This study	https://github.com/vclabsysbio/scRNAseq_DVtimecourse

**Software and algorithms**		

RStudio v3.1.4	RStudio	https://www.rstudio.com/
FastQC v0.11.9	Babraham Bioinformatics, 2010	https://www.bioinformatics.babraham.ac.uk/projects/fastqc/
Cell Ranger v3.0.2	10x Genomics	https://www.10xgenomics.com/
Seurat v3.1.2	(Stuart et al., 2019)	https://satijalab.org/seurat/
SoupX v1.0.1	(Young and Behjati, 2020)	https://github.com/constantAmateur/SoupX
DoubletFinder v2.0.3	(McGinnis et al., 2019)	https://github.com/chris-mcginnis-ucsf/DoubletFinder
Monocle3 v0.2.3.0	(Cao et al., 2019)	http://cole-trapnell-lab.github.io/monocle-release/monocle3/
gProfier2 v0.1.8	(Raudvere et al., 2019)	https://biit.cs.ut.ee/gprofiler/
ggplot2 v3.3.2	(Wickham, 2016)	https://ggplot2.tidyverse.org/
ComplexHeatmap v2.4.3	(Gu et al., 2016)	https://github.com/jokergoo/ComplexHeatmap
FlowJo v10.7.1	TreeStar Inc	https://www.flowjo.com/

### Resource availability

#### Lead contacts

Further information and requests for resources should be directed to and will be fulfilled by the lead contact: Varodom Charoensawan (varodom.cha@mahidol.ac.th).

#### Materials availability

This study did not generate new unique reagents.

### Experimental model and subject details

#### Human subjects and ethics approval

The study was approved by the Institutional Review Boards of Faculty of Medicine Vajira Hospital (No.015/12), Faculty of Tropical Medicine Mahidol University (TMEC 13041) and Faculty of Medicine, Ramathibodi Hospital, Mahidol University (MURA2019/603). As part of the DENFREE initiative (https://cordis.europa.eu/project/id/282378/results) (Matangkasombut et al., 2020), we obtained the peripheral blood mononuclear cells (PBMCs) from two Thai male adult donors, both diagnosed with secondary DENV-4 infection with DF and DHF severities, aged 35 and 20 years old, and the viral loads of 1.76 x 10^6^ and 1.77 x 10^7^, respectively (Table S16). The PBMC samples were collected at the defervescence (“Def”) day, two and one days before Def (“Day 2” and “Day 1”), also known as the days of febrile illness, and two weeks after Def (“Wk2”), which was considered as convalescence or follow-up (see Figure 1A).

### Method details

#### PBMC isolation

After plasma collection, cell suspensions were diluted in the RPMI 1640 medium (Gibco™, USA) supplemented with 2% fetal bovine serum (FBS) (Invitrogen, USA) before isolation on Isoprep, an isolation medium for separation of human lymphocytes (Robbins Scientific Corporation, USA). Cells were washed and resuspended in the RPMI 1640 medium (Gibco™, USA) completed with 0.5% FBS. The PBMC samples were stored in cryopreservative reagents containing 90% FBS (Invitrogen, USA) and 10% Dimethyl sulfoxide (DMSO) (Sigma-Aldrich, USA) and kept in liquid nitrogen until use. For the controls, we included two independent healthy PBMCs to the analyses. “HCI” was stored and processed following the same protocols as the DENV samples, whereas “HCII” was a publicly available single-cell PBMC profile obtained from the 10x genomics’ online resource (as listed in the the key resources table).

#### Single-cell preparation and library construction

Frozen PBMCs were thawed, and dead cells were removed using the Dead Cell Removal kit (Miltenyi Biotec, Germany), resulting in the cell viabilities of 95% or higher in all the samples. Cells were resuspended in Dulbecco’s phosphate buffered saline (DPBS) (Gibco™, USA) supplemented with 0.04% bovine serum albumin (BSA) (Sigma-Aldrich, USA). Single-cell isolation and library preparation were performed using the Chromium Single Cell 3′ Reagent (v2), following the manufacturer’s protocols (10x Genomics, USA), with the expected cell numbers of 5,000 cells per sample. The quality of the single-cell libraries were assessed by the Agilent High sensitivity D1000 Tapestation (Agilent, USA), and Qubit (Thermo Fisher Scientific, USA), and sequenced through the sequencing service provided by Macrogen Inc., Korea, targeting 50,000 reads per cell. Raw reads are available in the ArrayExpress repository under the accession number E-MTAB-9467**.**

#### Pre-processing of scRNA-seq data

Sequenced data were assessed for their overall sequencing qualities using FastQC (www.bioinformatics.babraham.ac.uk/projects/fastqc), and analyzed using CellRanger version 3.0.2 (10x Genomics, USA) and the reference human genome GRCh38 1.2.0. The expression matrices containing the cell barcodes and transcript counts are also available from ArrayExpress (E-MTAB-9467), and the numbers of reads and assigned cells from each sample can be found in Table S18. In total, the combined scRNA-seq data set from eight DENV samples and a new healthy sample are 39,885 individual cells, with the average number of reads per cell of 57,528.

Data pre-processing, comprising data normalization, clustering and dimensionality reduction, were performed using Seurat V3 (Stuart et al., 2019). To normalise the transcript counts, regularized negative binomial regression via the *SCTransform()* function (Hafemeister and Satija, 2019) in Seurat V3 was used. Cell clusters were identified by the shared nearest neighbor (SNN) method using the Louvain algorithm with the resolution of 0.8 (default settings), and the first 30th principal components (PCs). The dimensionality reduction was performed using the *RunUMAP* function, also on the first 30th PCs.

To remove potential contamination of ambient RNAs, SoupX (Young and Behjati, 2020) was applied to each sample before data integration. Immunoglobulin (Ig) genes that were not expected to be expressed in certain cell types and clustering information were provided as inputs to estimate the contamination fraction. The expression values were then adjusted from the initial count matrices. Cells expressing mitochondrial genes of 10% or more of the total reads were excluded. Doublets and multiplets were also discarded using doubletFinder (McGinnis et al., 2019) with the default settings, and the “pK” value of each sample set at the maximal value of mean-variance normalized bimodality coefficient. The numbers of remaining cells after quality control steps were provided in Table S19.

#### Data integration and normalization

After ambient RNA and doublet removal, scRNA-seq profiles of individual samples were further processed following the same pipeline for normalisation, clustering, and dimensional reduction as mentioned above. Then, the ten samples (eight DENV and two healthy control scRNA-seq profiles) were integrated using Seurat V3 (Stuart et al., 2019) using 3,000 gene features and Louvain algorithm with multi-level refinement, and other default settings were kept otherwise. We then re-clustered the integrated scRNA-seq profile using the clustering resolution of 3 (which gave the best clustering that matched the characterized cell types). Gene expression level of each cell was normalized using the function *NormalizaData,* where the unique molecular identifier (UMI) counts of each gene were divided by the total number of UMIs per cell, multiplied by scaling factor (10,000) and log-transformed.

#### Analyses of cell types and subpopulations

Clusters of cells were identified using characterized positive and negative marker genes as summarized in Table S1 and Figures S1, S6, and S19. We then re-integrated and re-clustered the subpopulations of different cell types separately using the same pipelines and settings as mentioned above, except for the resolutions, which were 5 and 3 for T and B cells, respectively.

#### Gene expression analyses

The list of highly variable genes (“HVGs”) across four time points of DENV infection were obtained from the union of the top 500 genes representing the first and second Principal Components (PCs) of average transcript levels of the “pseudo-bulk” RNA-seq of all cells. Pearson correlation coefficients and the Principal Component Analysis (PCA) were performed (*e.g.,* Figures 2A and S2) using the *cor()* and *prcomp()* functions in R, respectively. (Dis)similarities between objects were calculated using the *parDist()* function in R with the Dynamic Time Warping (DTW) method. Hierarchical clustering of HVGs was then performed using the Ward methods. The PC plots and heatmaps were produced using the ggplot2 (Wickham, 2016) and ComplexHeatmap R packages (Gu et al., 2016) (*e.g.,* Figures 2A and 2B), respectively. The genes with high levels of expression in certain subpopulations as compared to all other cells (*e.g.,* Figures 3C and 5C, also known as differentially expressed genes or “DEGs”) were identified using *FindAllMarkers* in Seurat V3 (Stuart et al., 2019) with the default settings, except that min.pct of 0.25 was specified, and only the positive markers were reported.

#### Data visualization and pseudotime analyses

Uniform Manifold Approximation and Projection (UMAP) and violin plots were generated using the *DimPlot* and *VlnPlot* functions in Seurat V3 (Stuart et al., 2019), respectively. The expression of genes or gene modules on the dimensional reduction was displayed using the *FeaturePlot* function (*e.g.,* Figures 1C and 2D). The dotplots that represent the average log-normalized expression of each gene and the percentages of cells that express more than one transcript were generated using the *DotPlot* function, also in Seurat V3 (*e.g.,* Figures 3D and 5D). Stacked bars, PCA, dotplots, boxplots were constructed using ggplot2 in R (Wickham, 2016).

To estimate the transition stage of cells from one functional stage to another stage, the single-cell trajectory analysis was performed on the T and B subpopulations in the DF and DHF samples using Monocel3 (Cao et al., 2019). Heamaps were produced using the *DoHeatmap* function in R. The plots of cells along trajectories were produced using the *plot_cells* function. The effector CD8-3 and naive CD4 T cells were set at the root of the pseudotime plots for the CD8 and CD4 T cell population, respectively, whereas naive B cells were set as the root of the B cell pseudotime plot. The *plot_cells* command was used for trajectory visualisation.

#### Pathway analyses and gene expression scoring

g:Profiler2 (Raudvere et al., 2019) was used to assess the functional enrichment of different gene groups with unique expression patterns (*e.g.,* Figures 2B–2D), using the reference human genes from the annotation version GRCh38 1.2.0 as the background. Benjamini-Hochberg FDR was applied for the multiple testing correction and computing adjusted p-values, where the significance threshold was at 0.05. The same BP analyses were also performed for each subpopulation (Figure S3, and Tables S6, S7, S8, S9, S10, and S11). Signature genes of each BP were then scored for individual cells using the *AddModuleScore* function (Figures 2D and S4) from Seurat V3 (Stuart et al., 2019). We visualized the relative expression of all the HVGs belonging to different BPs (see Table S11) in the four major cell types (*i.e.,* monocytes, natural killer, T and B cells) by computing the average expression of a HVG in a particular celltype, divided by the average expression of that HVG in all the four cell types (pseudocount of 1 was added) (Figure 2C). The sum of expression of all the HVGs in a particular BP was then z-score transformed across the four cell types and time points to reflect the relative expression of the genes in the major BPs.

#### Flow cytometry

The PBMC samples were recovered and stained against the following antibodies and their respective isotype controls: Anti-CD3-PerCP, Anti-CD4-BV510, Anti-CD8-APCCy7, CD69-BV421, PD-1-Alexa700, CLA-PE and CD103-APC (all from Biolegend, USA, see also the key resources table). The cells were fixed by 1% paraformaldehyde (Sigma-Aldrich, USA) and analysed using CytoFlex (Beckman Coulter, USA). The data were exported and analysed by Flowjo v10.7.1 (TreeStar Inc, USA). Percentages of “cell positive” were normalized by the isotype controls of the same donors and time points.

### Quantification and statistical analysis

The statistical significance of the differences between the percentages of the CD8^+^ and CD4^+^T cells expressing the surface protein markers of interest between two given time points of the same patients was calculated using the one-tailed Wilcoxon signed-rank test. For the differences across more than two samples, Kruskal-Wallis, followed by Dunn’s test with the Benjamini-Hochberg method for multiple comparisons were applied (significance levels, ns = p > 0.05, ∗p ≤ 0.05, ∗∗p ≤ 0.01, and ∗∗∗p ≤ 0.001) (Figures 4, S16, and S18). The number of samples included (exact value of n) in each experiment can be viewed in the figure legends.

## Data Availability

• Single-cell RNA-seq data of the DENV patients and a healthy Thai donor generated in this study have been deposited at ArrayExpress and are publicly available as of the date of publication. Accession number is listed in the key resources table. • All computer codes used for the analyses have been deposited at GitHub and are publicly available as of the date of publication. The Github link is listed in the key resources table. • Any additional information required to reanalyze the data reported in this paper is available from the lead contact upon request.

## Supplemental information

Document S1. Figures S1–S25

Table S1. Established canonical markers for immune celltype identification used in this study, related to Figures 1B, 1C, 3Am S1,S6, S18 and STAR Methods

Table S2. Numbers and percentages of immune cell types in each scRNA-seq profile, related to Figures 1C, 3B, and 5A

Table S3. Average expression of the top 500 genes in Principal Component (PC) 1 and PC2, related to Figure 2A and 2B

Table S4. List of genes in each overall heatmap cluster, related to Figure 2B

Table S5. Biological process GO terms of each overall heatmap cluster, related to Figure 2B

Table S6. Average expression of the top 500 genes in Principal Component (PC) 1 and PC2 in monocytes, related to Figures 2C, S3 and STAR Methods

Table S7. Average expression of the top 500 genes in Principal Component (PC) 1 and PC2 in NK cells, related to Figures 2C, S3 and STAR Methods

Table S8. Average expression of the top 500 genes in Principal Component (PC) 1 and PC2 in T cells, related to Figure 2C, S3 STAR Methods

Table S9. Average expression of the top 500 genes in Principal Component (PC) 1 and PC2 in B cells/Plasma cells/Plasmablasts, related to Figure 2C, S3 and STAR Methods

Table S10. Percentages of cells in populations of interest that expressed particular genes, related to Figures 2C, S3 and STAR Methods

Table S11. List of biological processes of the “highly variable genes” (“HVGs”) from the four major immune cells, related to Figures 2C, 2D, and STAR Methods

Table S12. Normalized gene expression of the genes belonging to each GO term of interest in monocytes, related to Figures 2C and 2D

Table S13. Normalized gene expression of the genes belonging to each GO term of interest in NK cells, related to Figures 2C and 2D

Table S14. Normalized gene expression of the genes belonging to each GO term of interest in T cells, related to Figures 2C and 2D

Table S15. Normalized gene expression of the genes in each GO term in B cells/Plasma cells/Plasmablasts, related to Figures 2C and 2D

Table S16. Viral loads of the DF and DHF patients at febrile period and defevescense, related to STAR Methods

Table S17. Percentages and numbers of γδ T and NK cells in different canonical marker genes, related to Figures 3A, 3C, and S11

Table S18. Numbers of reads and assigned cells in each scRNA-seq profile, related to STAR Methods

Table S19. Numbers of cells passing each quality control step, related to STAR Methods
