# Supplementary material for: Single-cell temporal analysis of natural dengue infection reveals skin-homing lymphocyte expansion one day before defervescence
Source: iScience. 2022 Mar 5;25(4):104034. doi: 10.1016/j.isci.2022.104034 (PMC8957021; doi:10.1016/j.isci.2022.104034)
Supplement: Document S1. Figures S1–S25 [file mmc1.pdf]

iScience, Volume *25*

Supplemental information

Single-cell temporal analysis of natural dengue infection reveals skin-homing lymphocyte expansion one day before defervescence

Jantarika Kumar Arora, Anunya Opasawatchai, Tiraput Poonpanichakul, Natnicha Jiravejchakul, Waradon Sungnak, DENFREE Thailand, Oranart Matangkasombut, Sarah A. Teichmann, Ponpan Matangkasombut, and Varodom Charoensawan

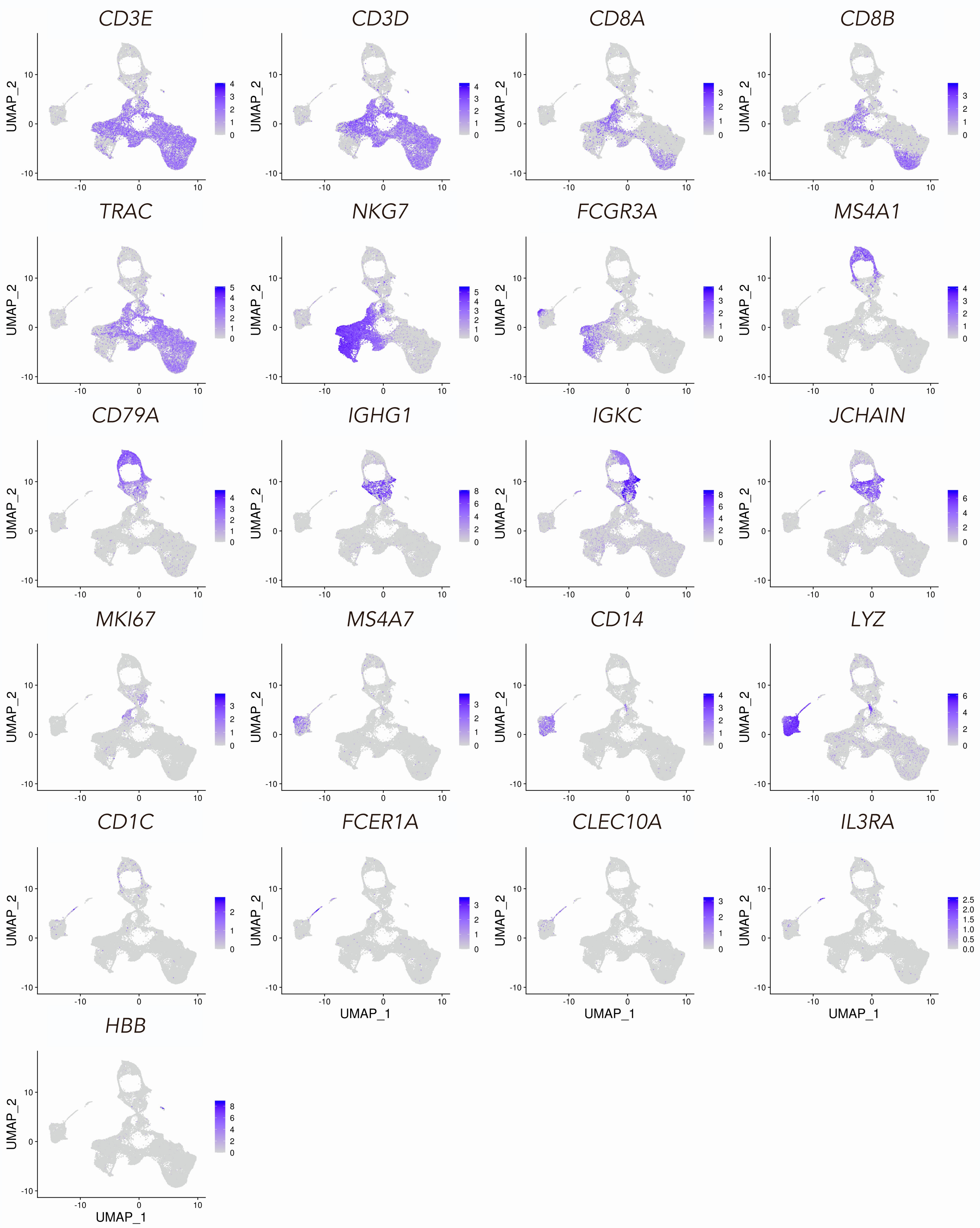

Figure S1. UMAP plots representing the expression of canonical markers of immune cell types. Related to Figure 1 and STAR Methods.

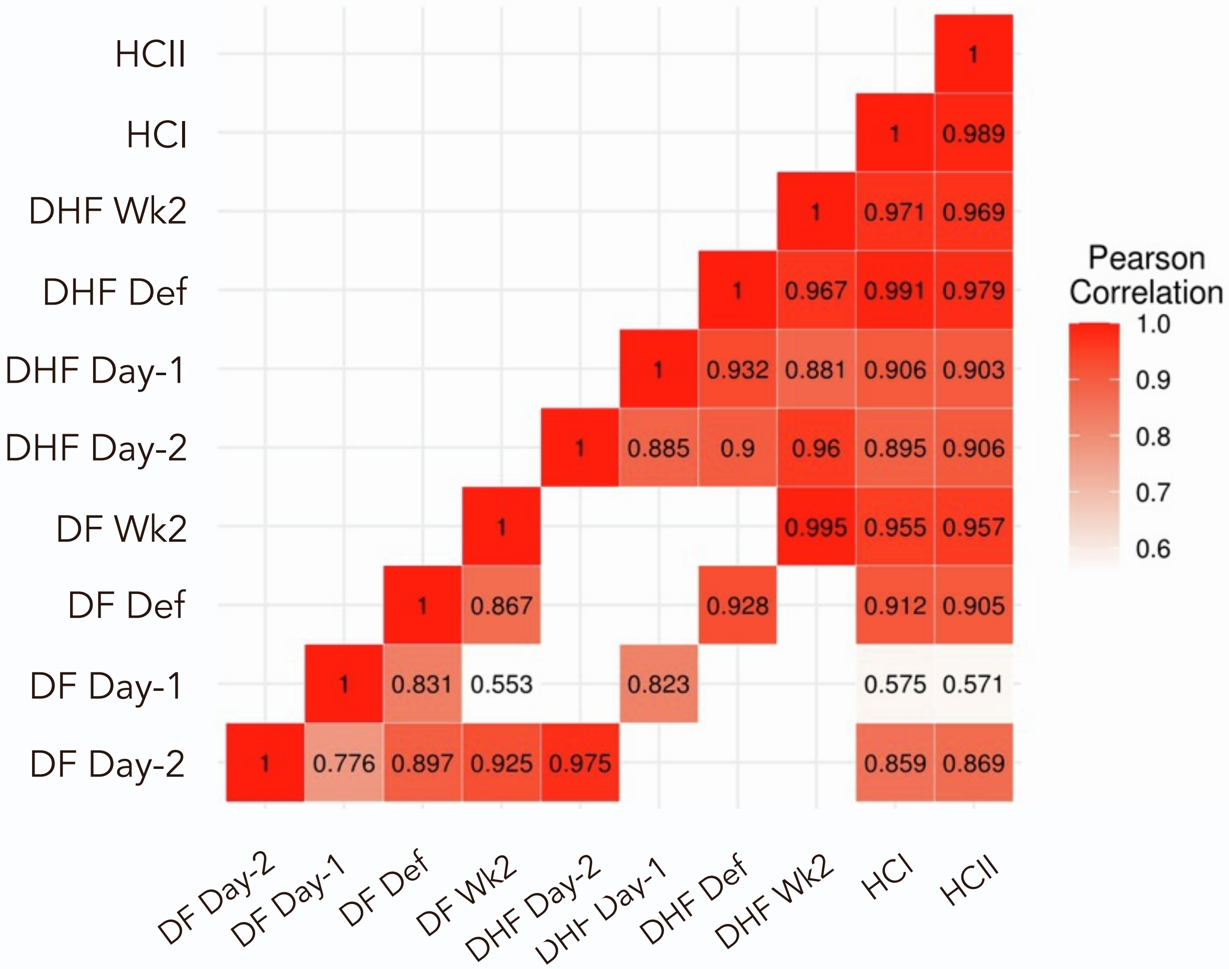

Figure S2. Pearson correlation coefficients of average gene expression in the PBMC samples. Related to Figure 2A and STAR Methods.

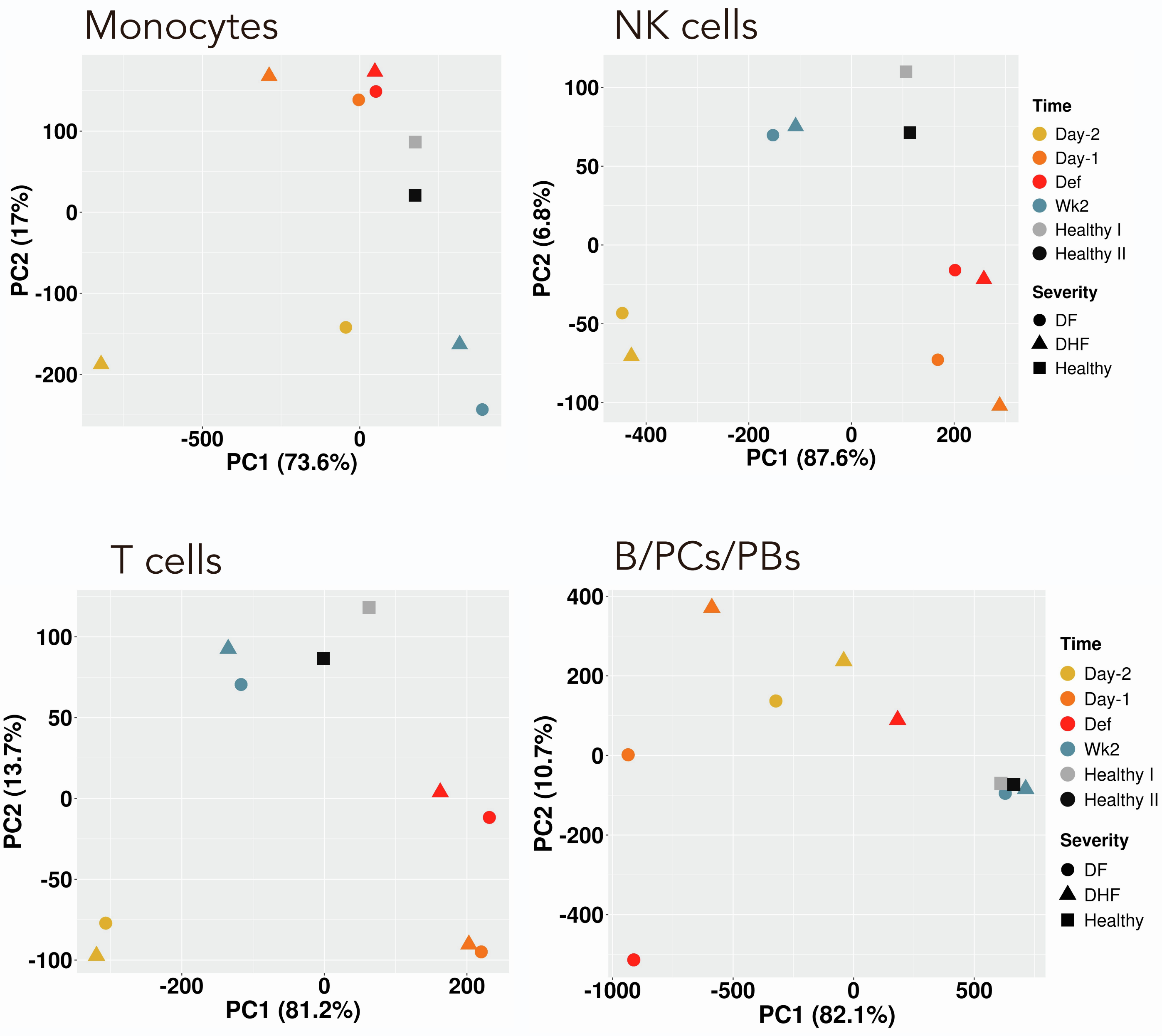

Figure S3. Principal Component Analysis (PCA) of average gene expression in monocytes, NK cells, T cells, and B cells/Plasma cells (PCs)/Plasmablasts (PBs). Related to Figure 2C and STAR Methods.

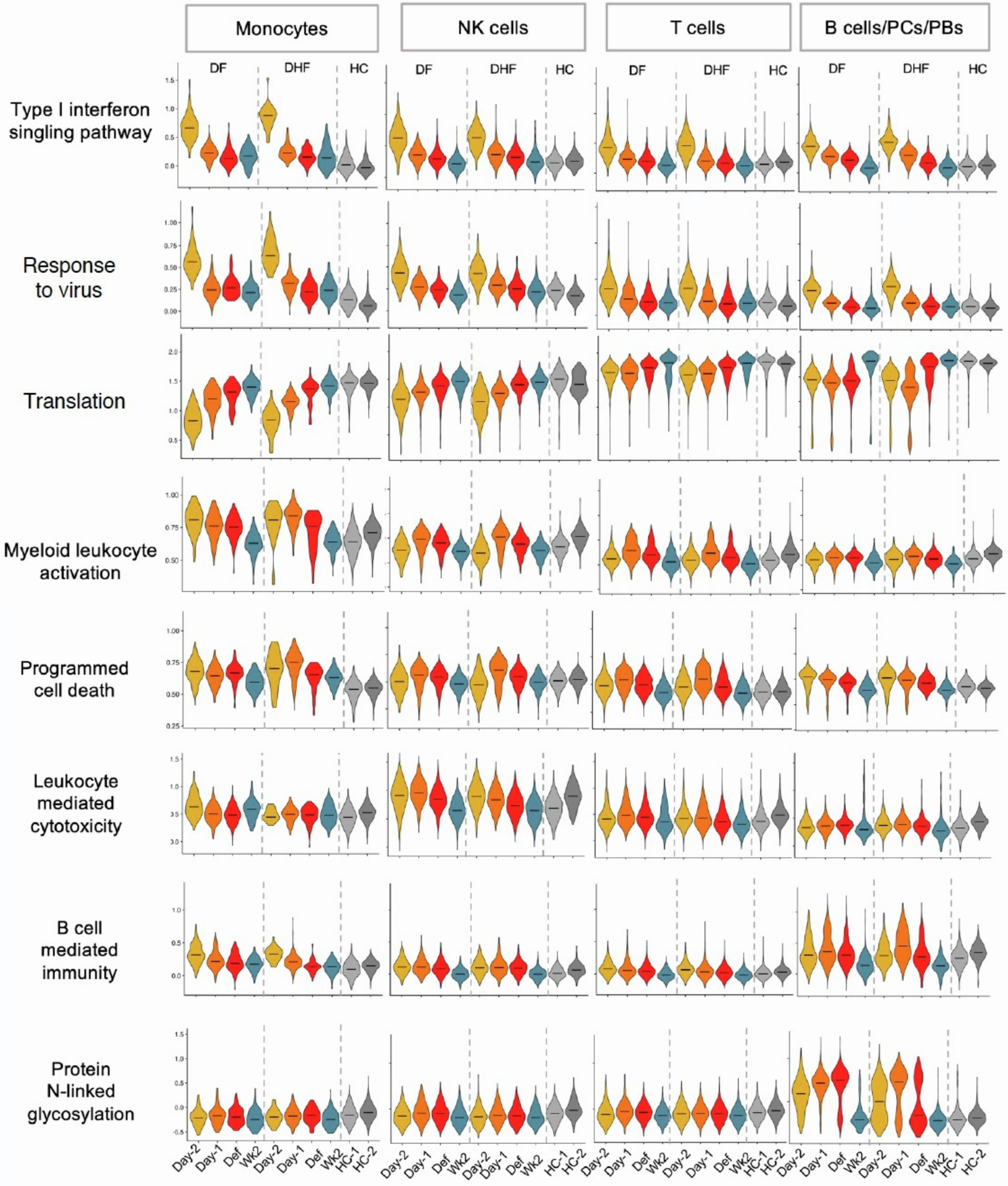

Figure S4. Violin plots representing the expression scores of the gene modules that are associated with each biological process over the course of DENV infection. Related to Figures 2C-D and STAR Methods.

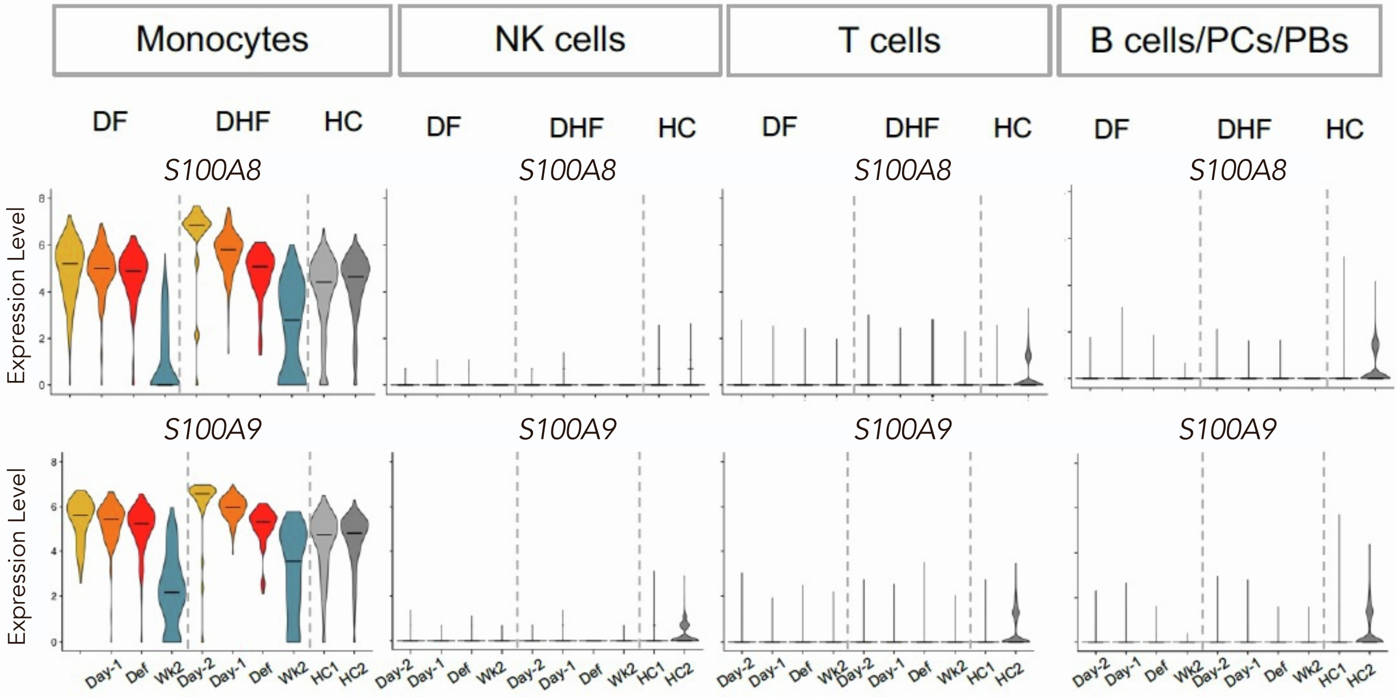

Figure S5. Violin plots representing the normalised expression of *S100A8* and *S100A9* over time courses of DENV infection. Related to Figure 2C-D.

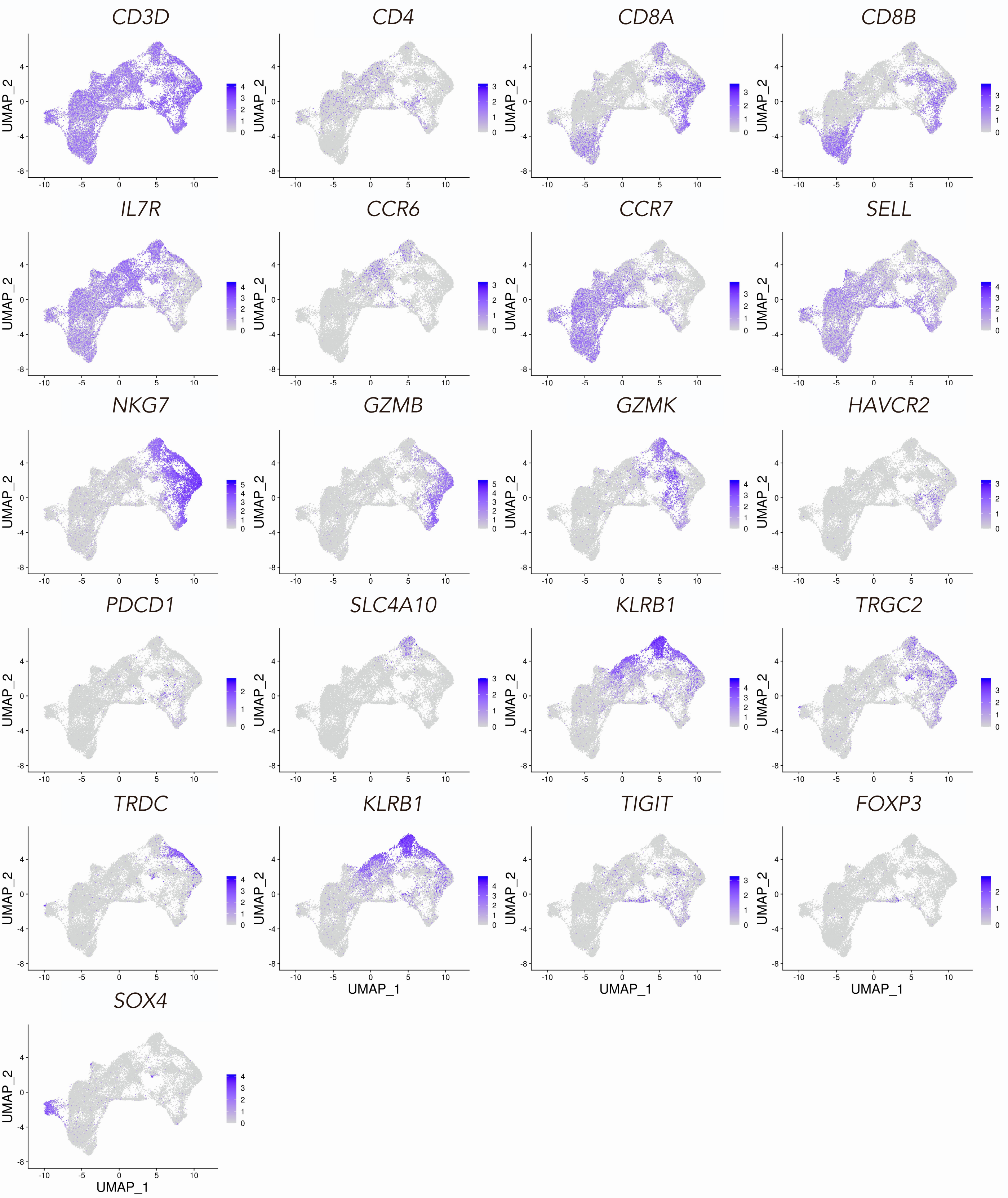

Figure S6. UMAP plots representing the expression of marker genes of T cell subpopulations. Related to Figure 3 and STAR Methods.

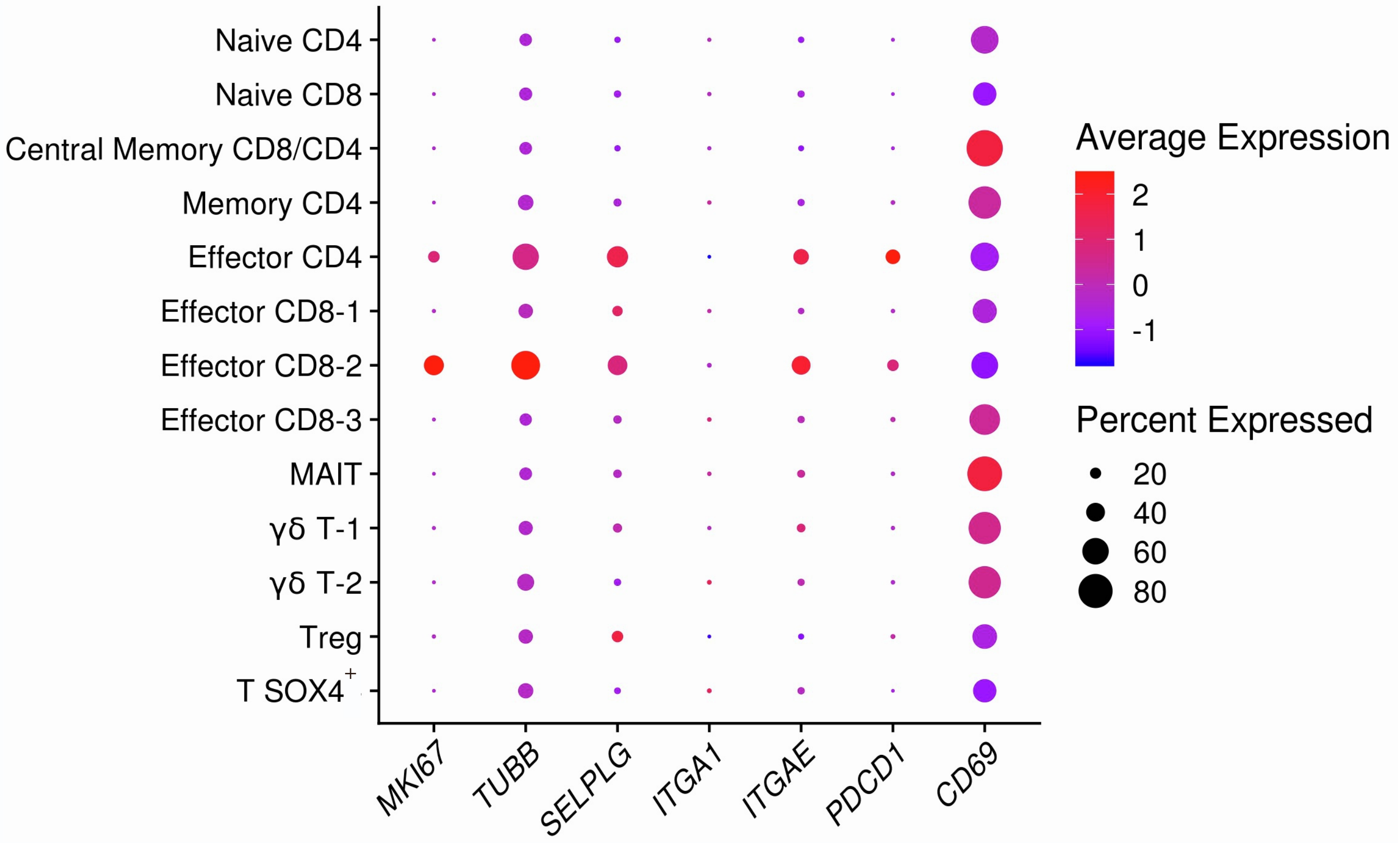

Figure S7. Dotplots representing the average gene expression of cells in certain populations. The dot sizes represent the proportion of cells expressing the genes. Related to Figure 3C.

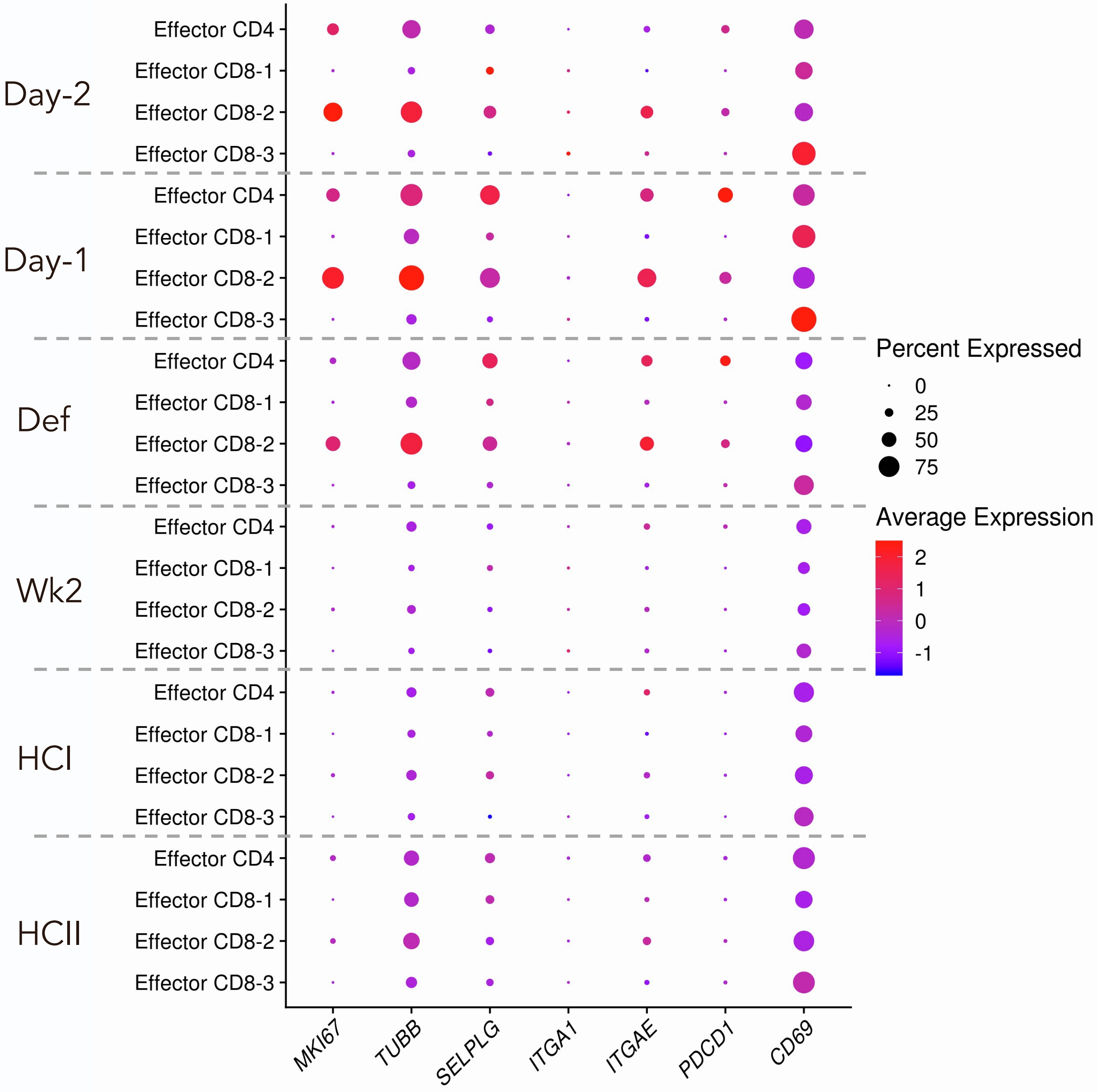

Figure S8. Dotplots representing the average gene expression and percentages of the effector CD4⁺ and CD8⁺ T cell subpopulations. The dot sizes represent the proportion of cells expressing the genes. Related to Figure 3C.

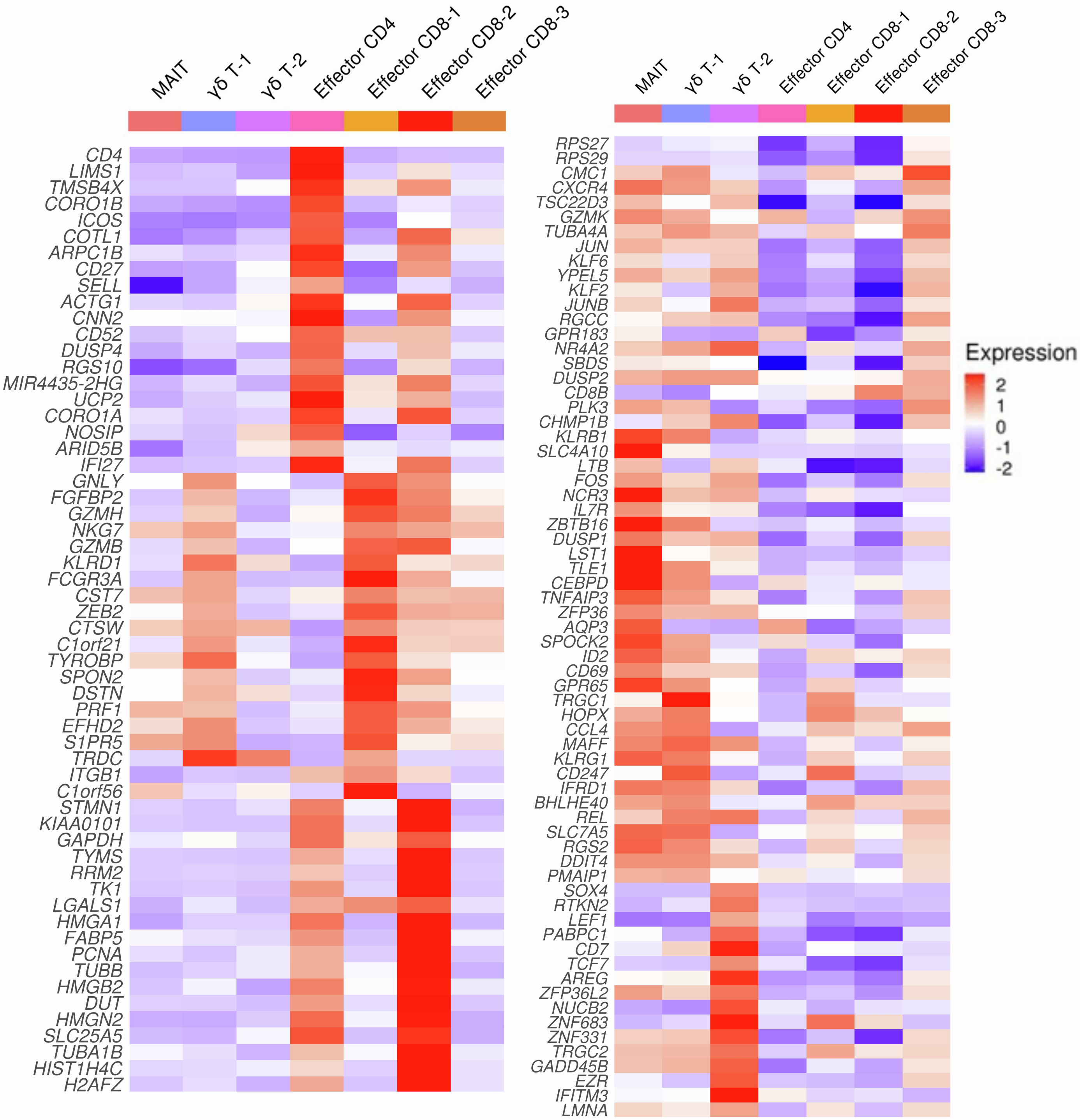

Figure S9. Heatmaps representing the relative expression of the top 20 genes from each of the effector T cell subpopulations. The genes of interest were selected from differentially expressed gene (DEG) analysis using the *FindAllmarkers* function (Stuart et al., 2019) across the effector-like subpopulations. Related to Figure 3C.

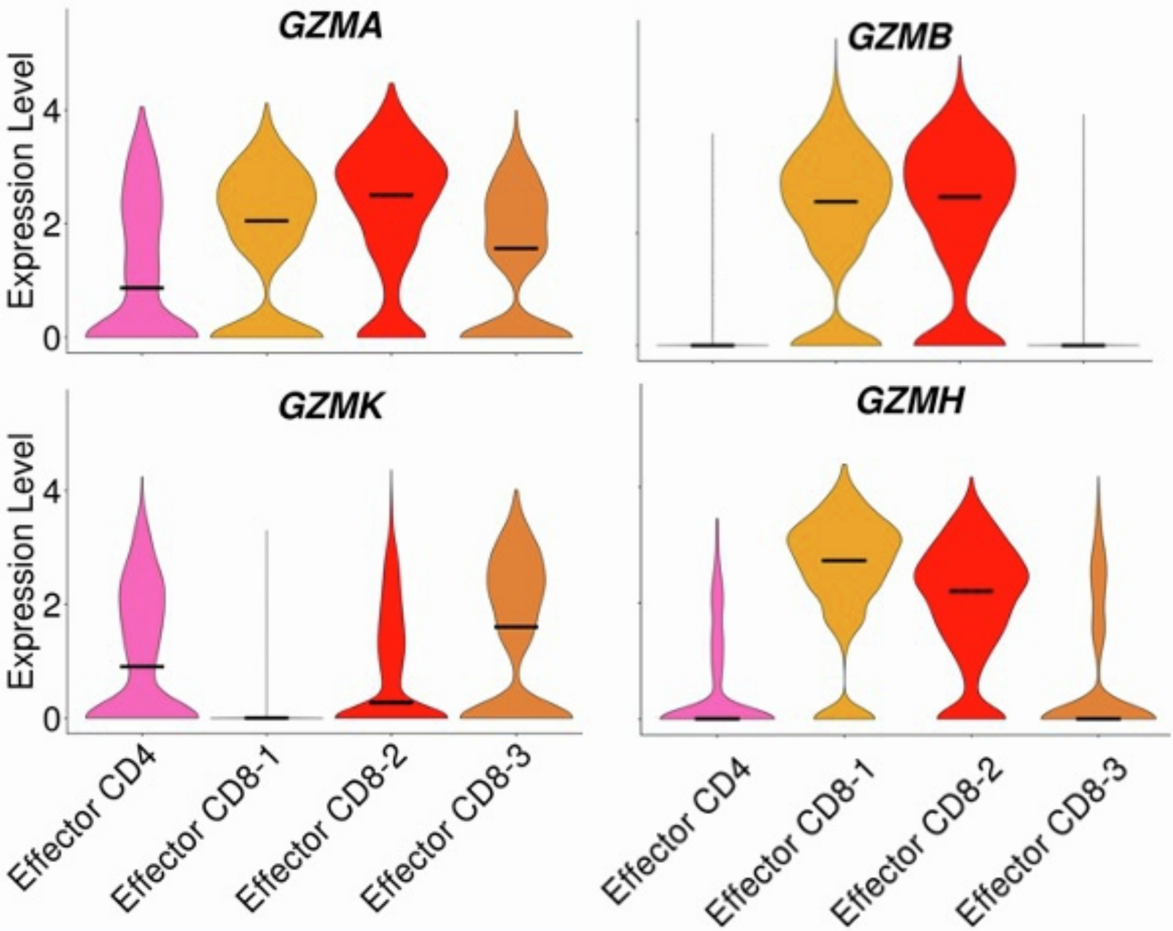

Figure S10. Violin plots representing the normalised expression of granzyme genes in the four subpopulations of effector T cells of interest. Related to Figure 3C.

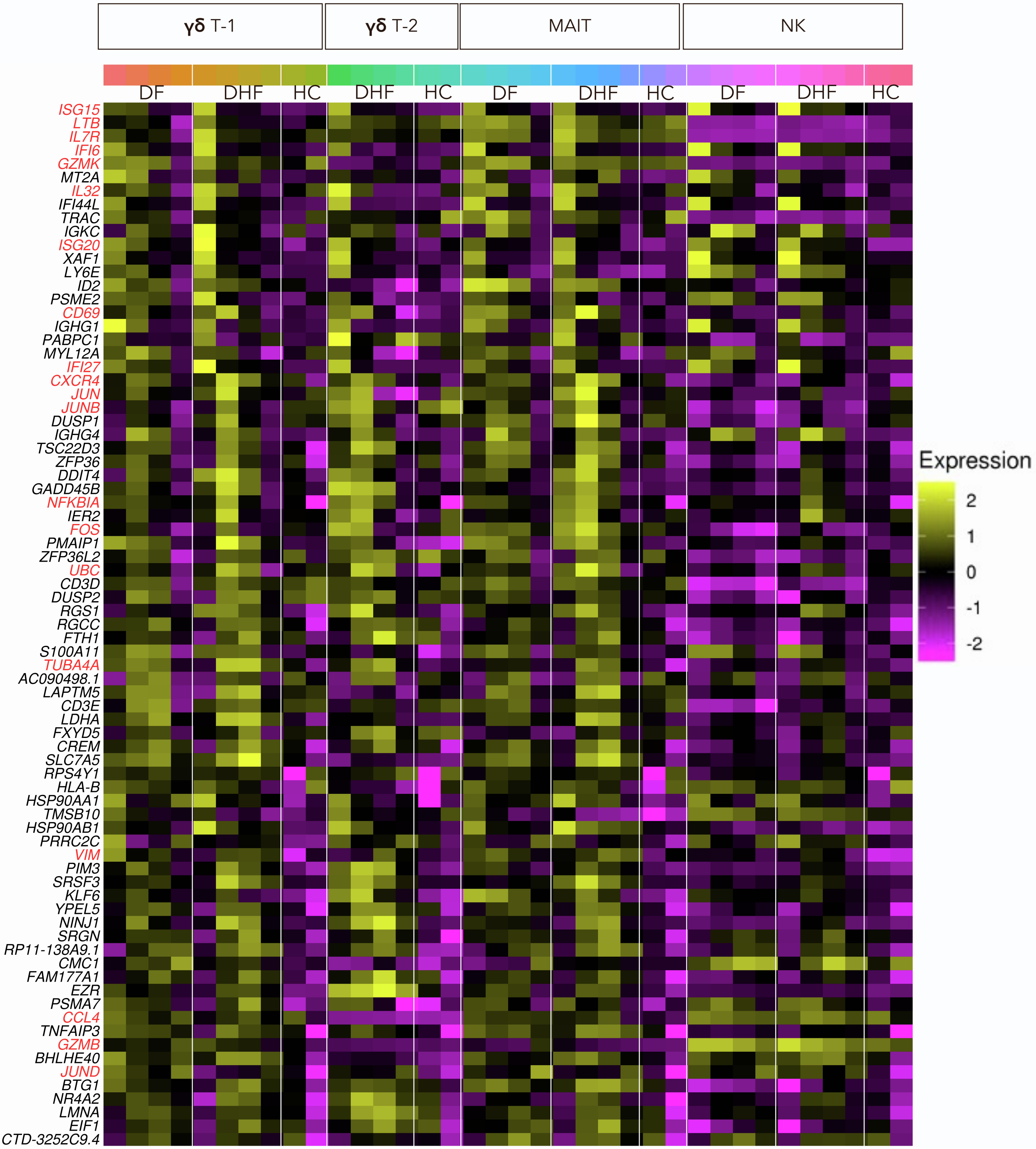

Figure S11. Heatmap representing the relative expressions of the top 20 genes from each acute day, compared to convalescence (Wk2). The genes of interest were selected by differentially expressed gene (DEG) analysis in the **γδ** T-1 population using the *FindMarkers* function (Stuart et al., 2019). Related to Figure 3C.

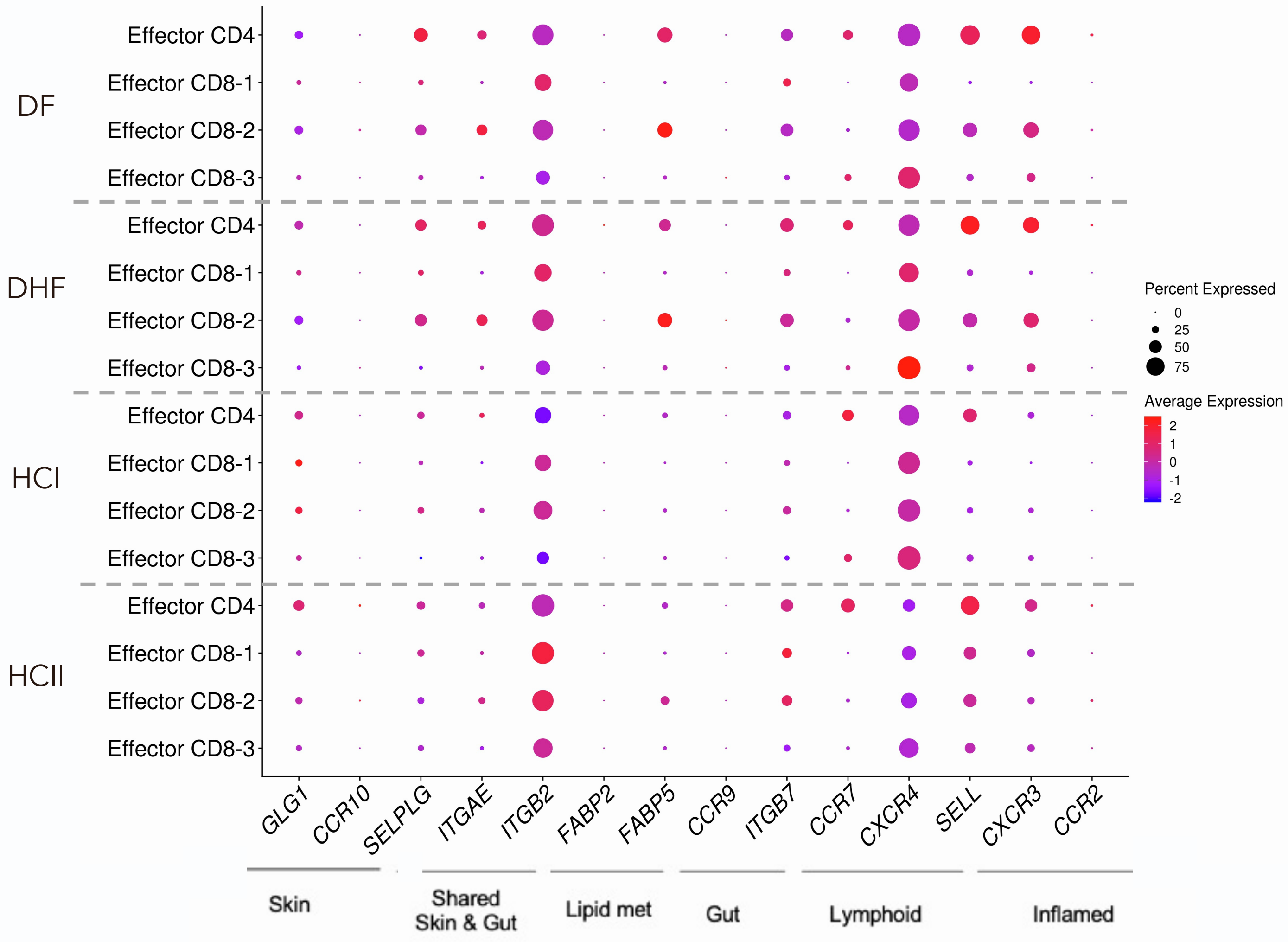

Figure S12. Dotplots representing average gene expression of the tissue-homing genes of interest in Effector T cell subpopulations. The dot sizes represent the proportion of cells expressing the genes. Related to Figure 3D.

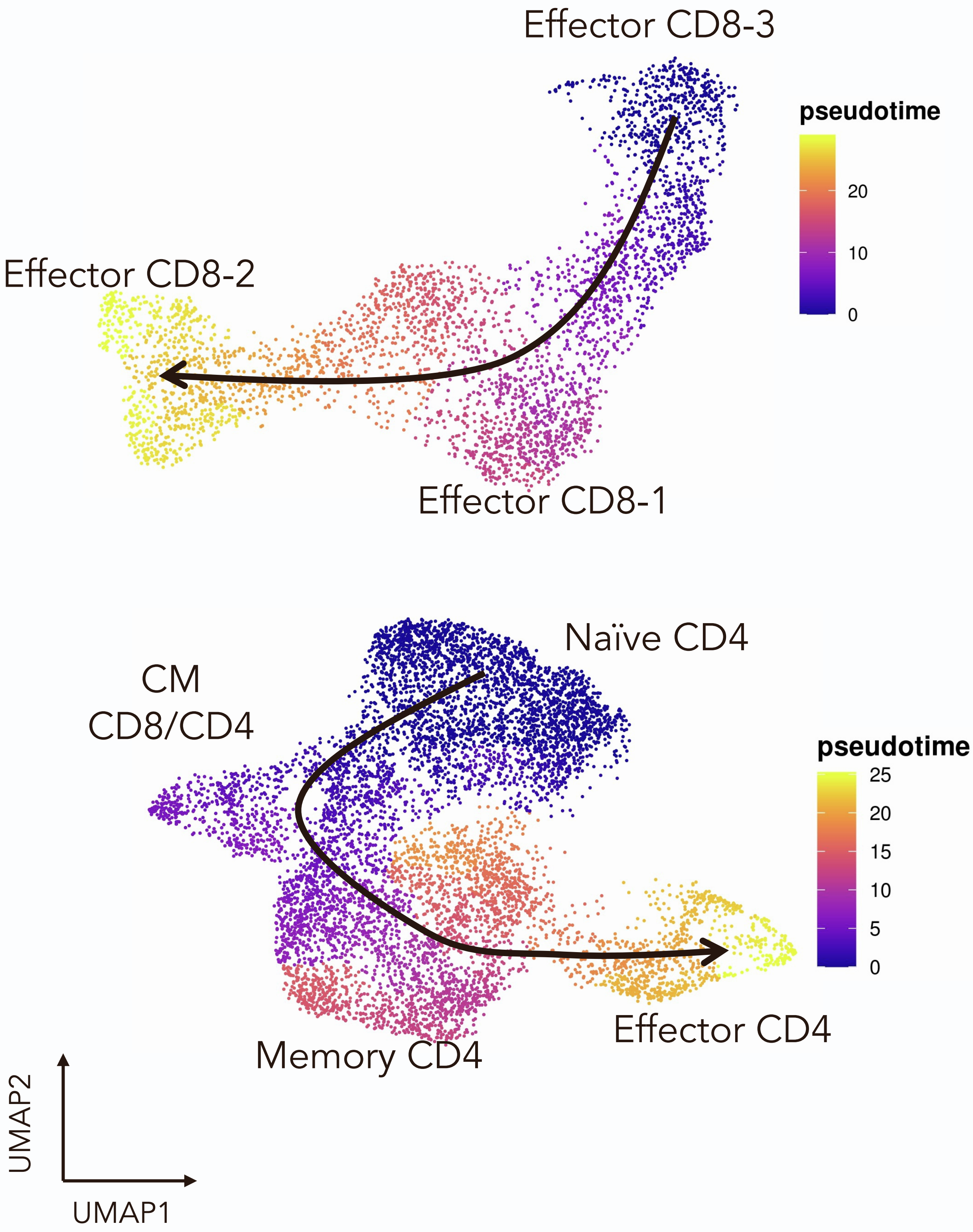

Figure S13. Pseudotime trajectory of three subpopulations of Effector CD8 T cells (top) and Effector CD4 T cells (bottom). Related to Figure 3E.

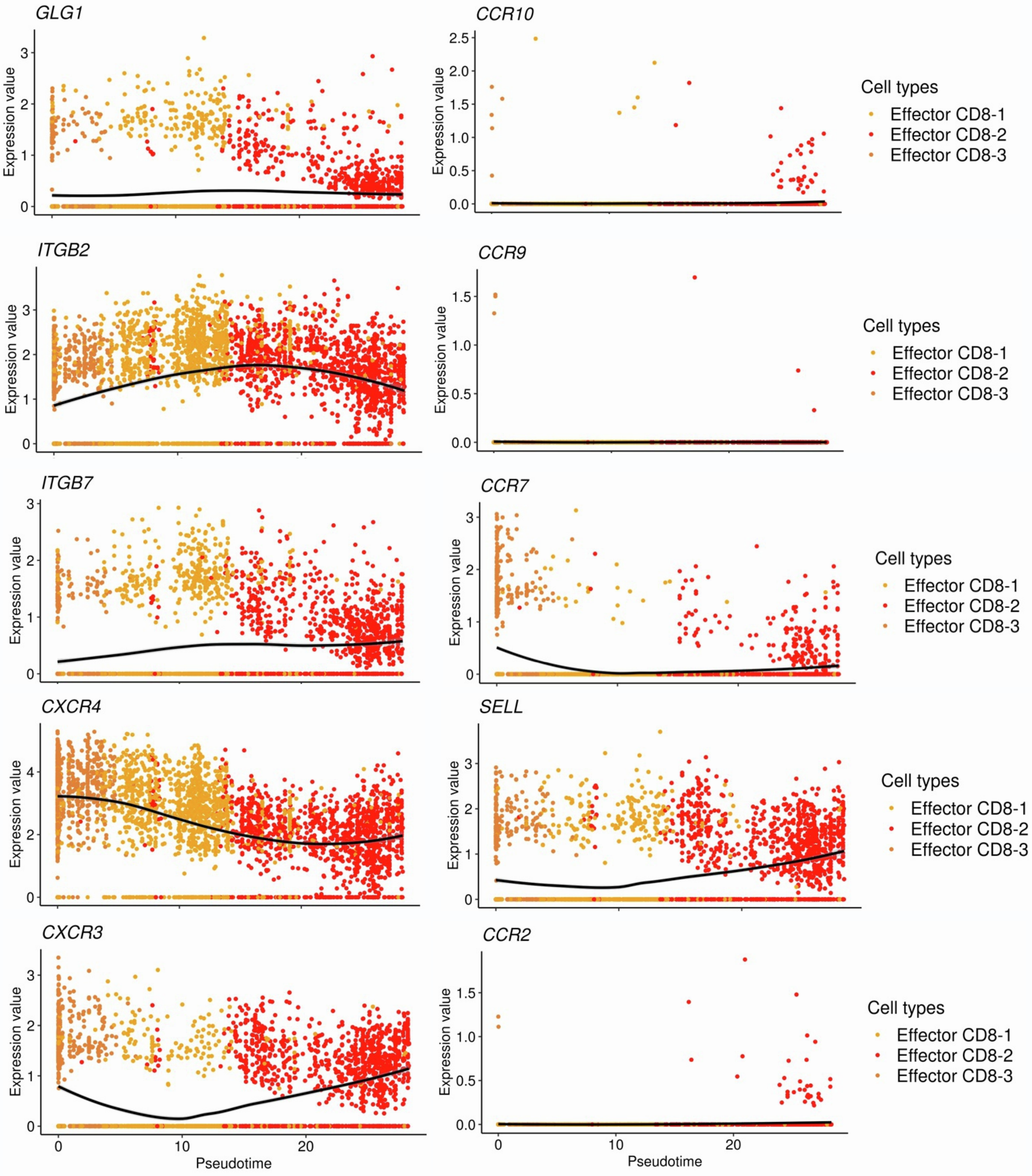

Figure S14. Expression of tissue-homing genes along the pseudotime in the effector CD8⁺ T cell subpopulations. Related to Figures 3E-F.

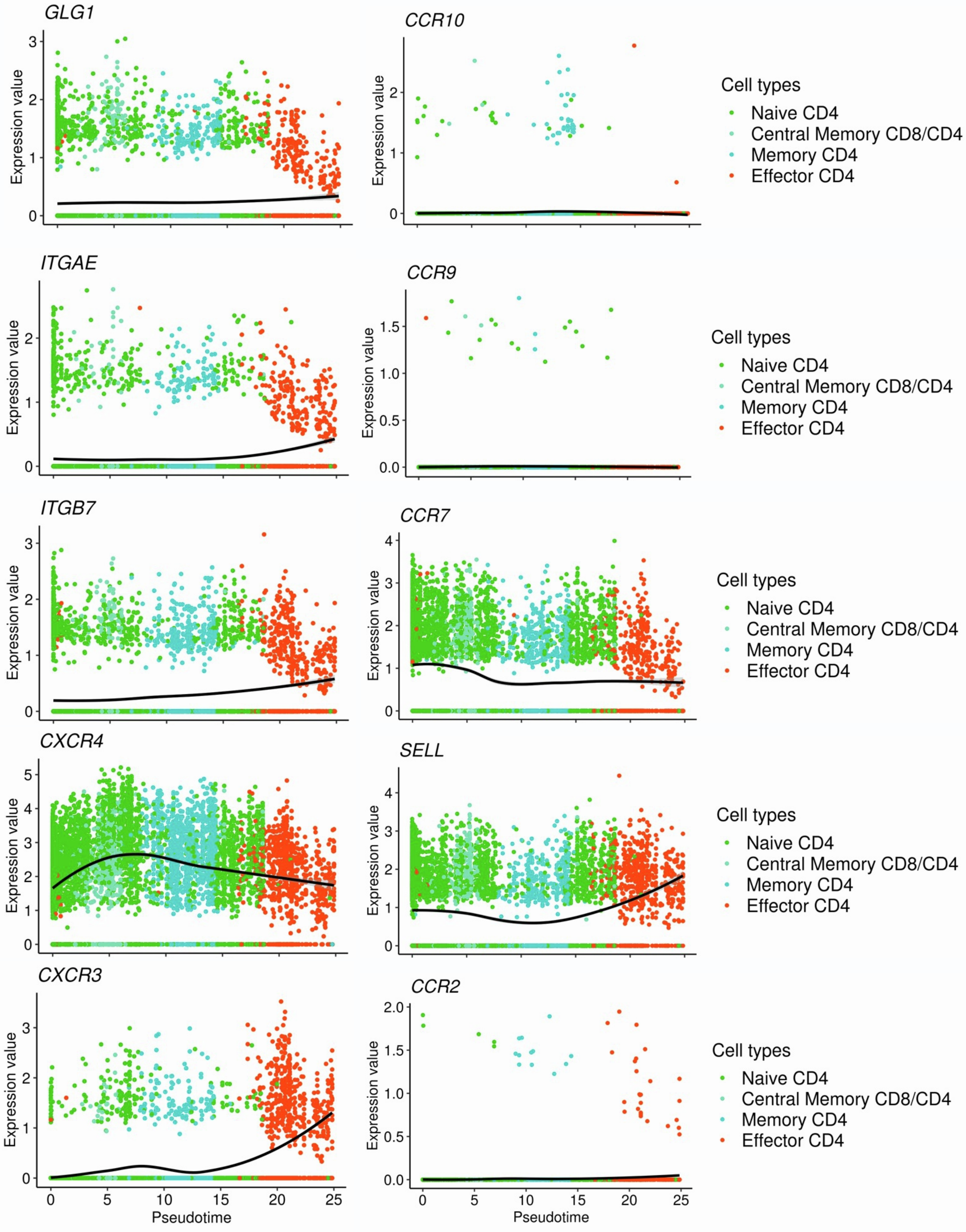

Figure S15. Expression of tissue-homing genes along the pseudotime in the effector CD4+ T cell subpopulations. Related to Figures 3E-F.

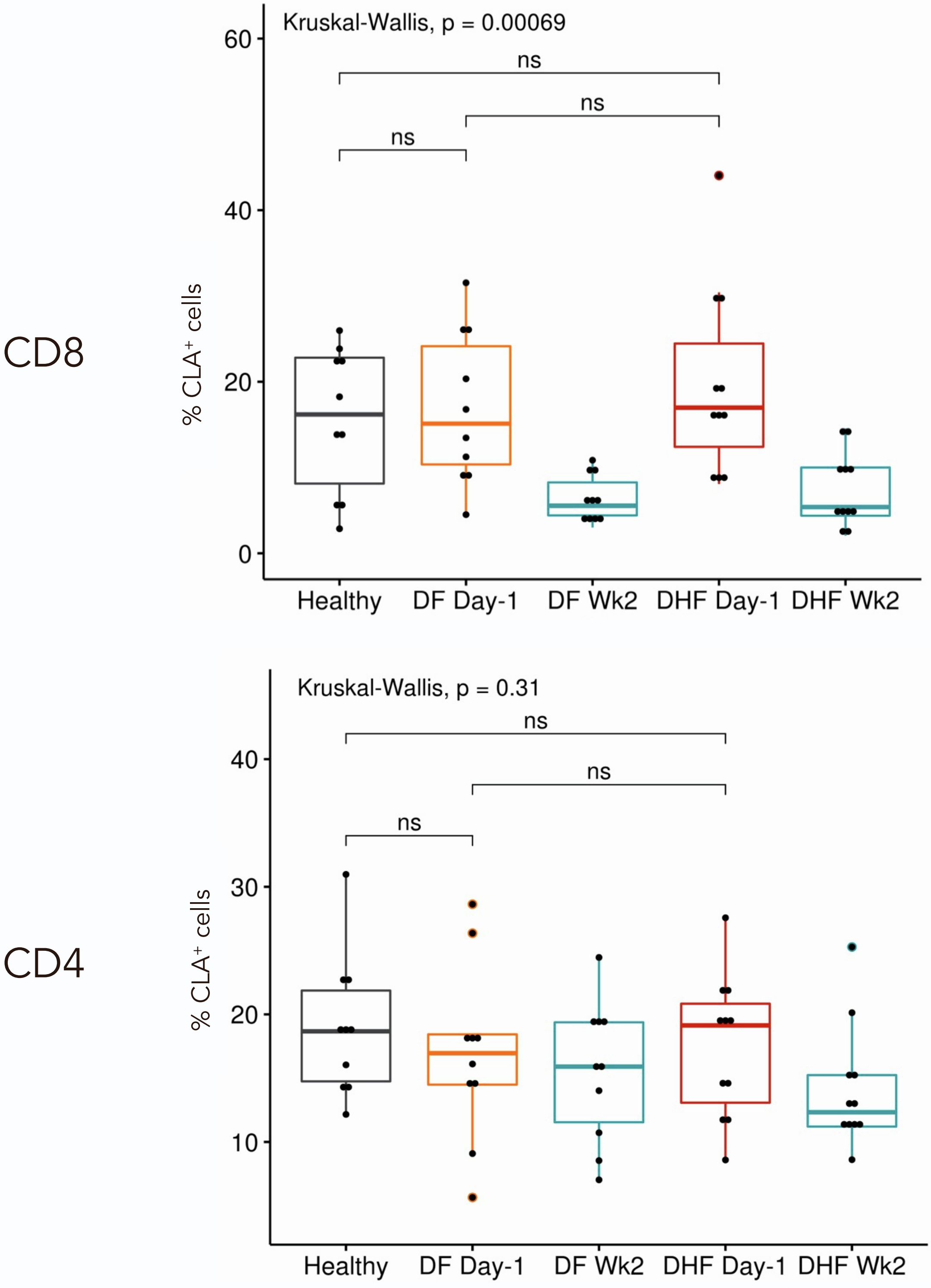

Figure S16. Box plots showing the percentages of the CLA^{+} cells within CD8^{+} (upper panel) and CD4^{+} T cell subpopulations (lower panel) based on protein expression from flow cytometry experiments. The Kruskal-Wallis test followed by Dunn's test with a Benjamini-Hochberg method was applied to compare the percentages of CLA^{+} cells. ns = $p > 0.05$, *$p \leq 0.05$, **$p \leq 0.01$, ***$p \leq 0.001$, and ****$p \leq 0.0001$. n = 10 for healthy controls, n = 10 for DF patients, and n = 11 for DHF patients. Related to Figure 4B and STAR Methods.

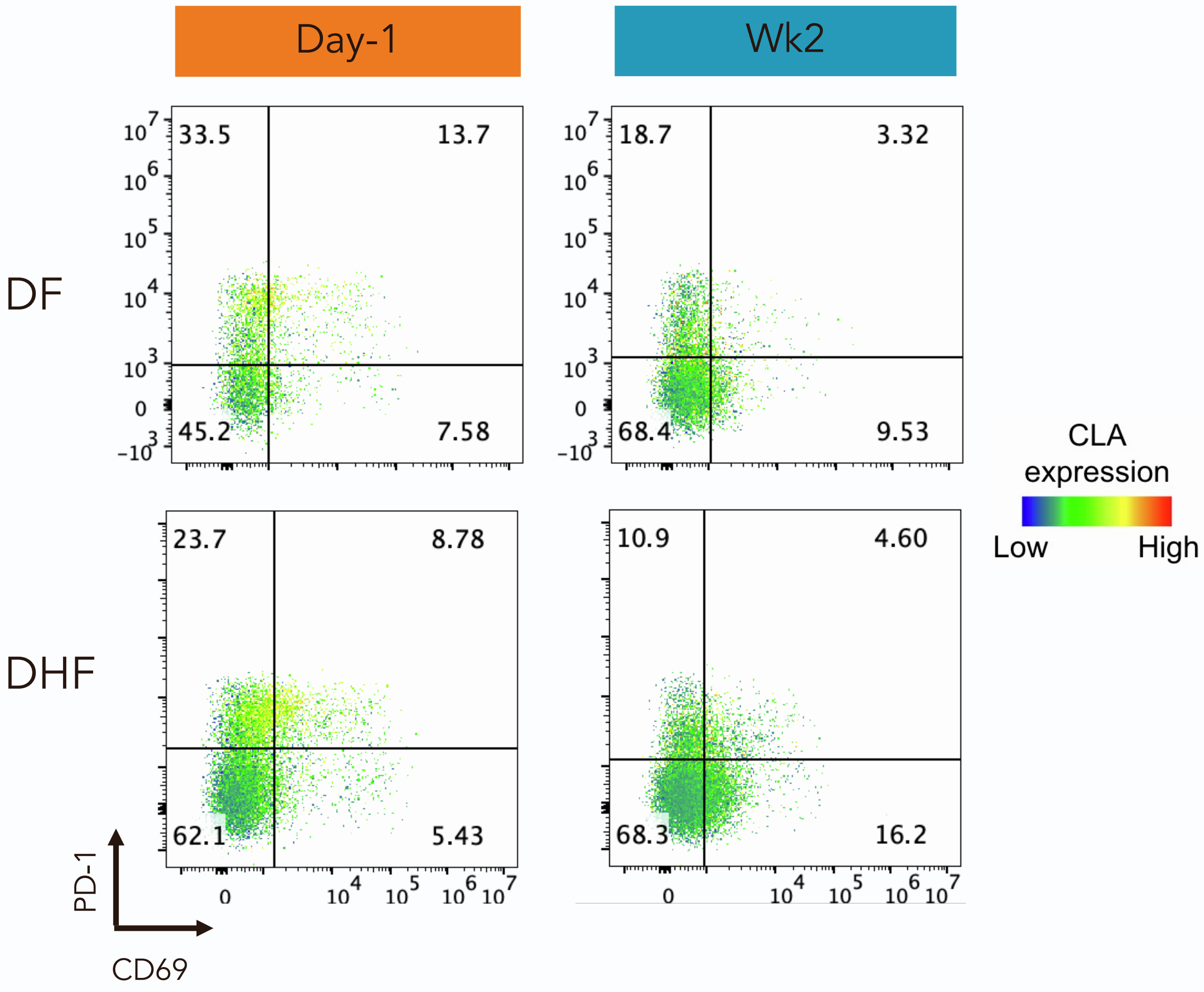

Figure S17. Relative expression levels of CLA in the CD69⁻ PD-1⁻, CD69⁺ PD-1⁻, CD69⁻ PD-1⁺ and CD69⁺ PD-1⁺ populations of the CD4 T cells. Related to Figure 4C.

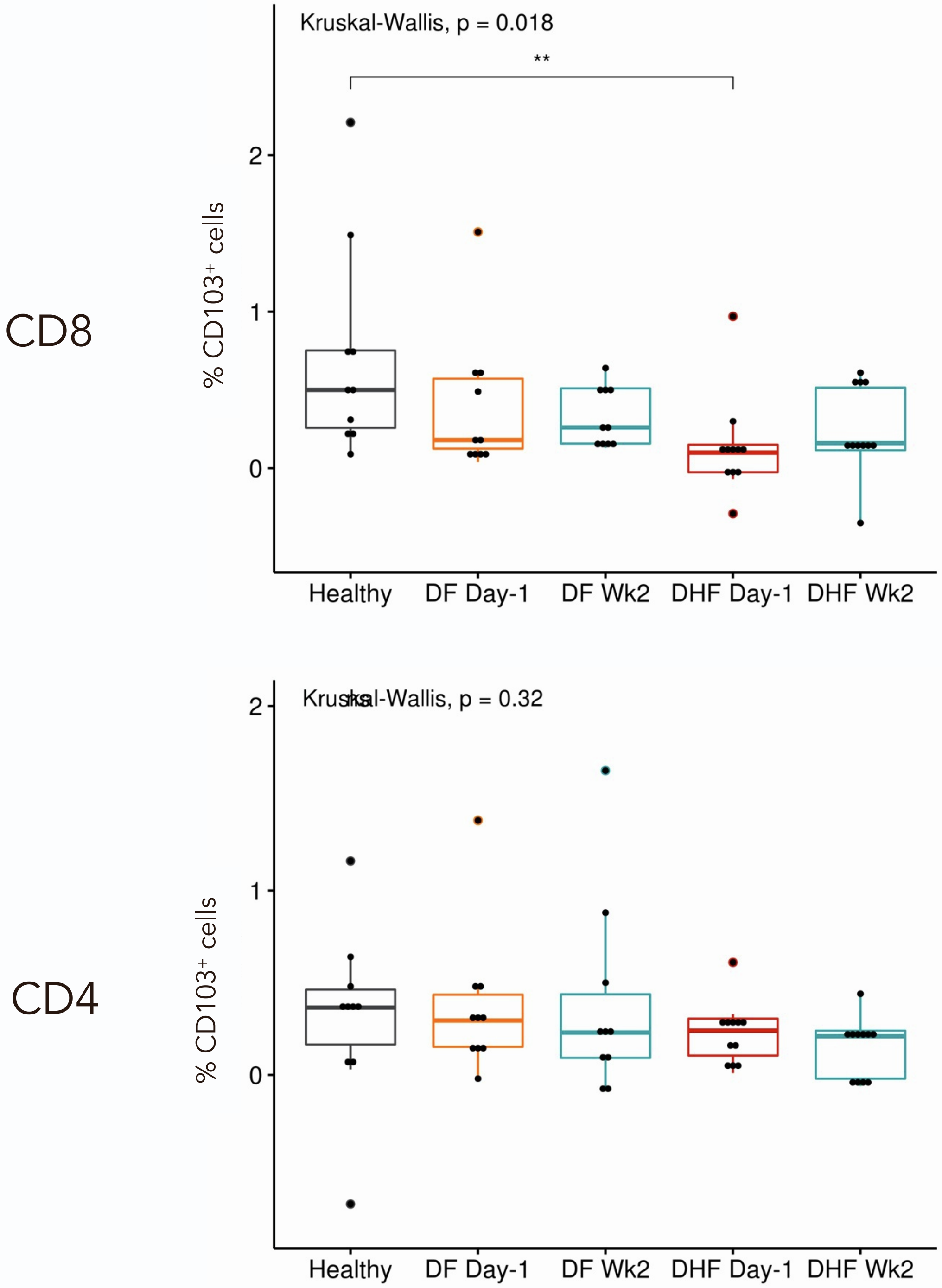

Figure S18. Box plots showing the percentages of the $CD103^{+}$ cells within $CD8^{+}$ (upper panel) and $CD4^{+}$ T cell subpopulations (lower panel) based on protein expression from flow cytometry experiments. The Kruskal-Wallis test followed by Dunn's test with a Benjamini-Hochberg method was applied to compare the percentages of $CD103^{+}$ cells. ns = $p > 0.05$, *$p \leq 0.05$, **$p \leq 0.01$, ***$p \leq 0.001$, and ****$p \leq 0.0001$. n = 10 for healthy controls, n = 10 for DF patients, and n = 11 for DHF patients. Related to Figure 4 and STAR Methods.

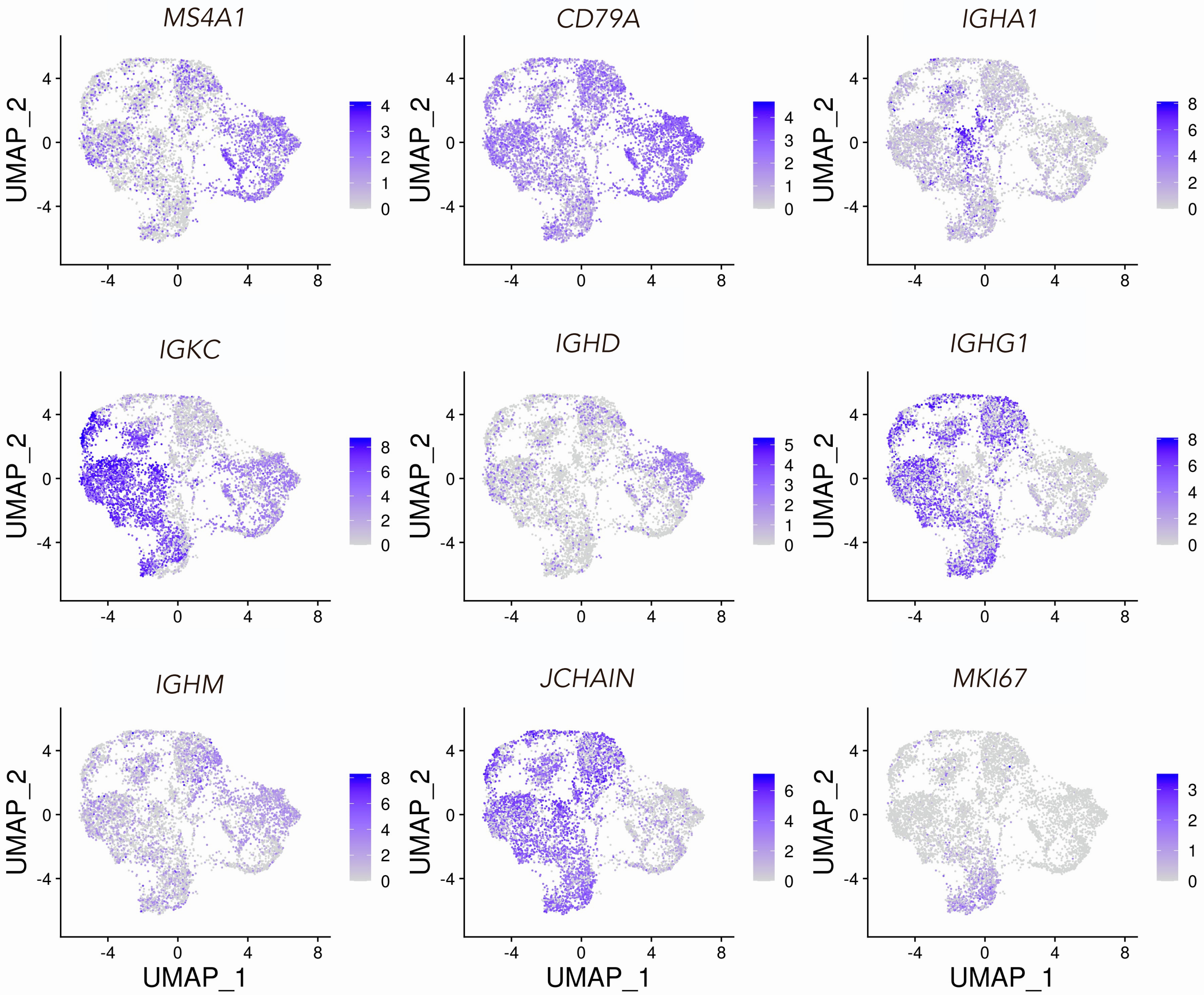

Figure S19. UMAP plots representing the expression of marker genes of the B cell subpopulations. Related to Figure 5 and STAR Methods.

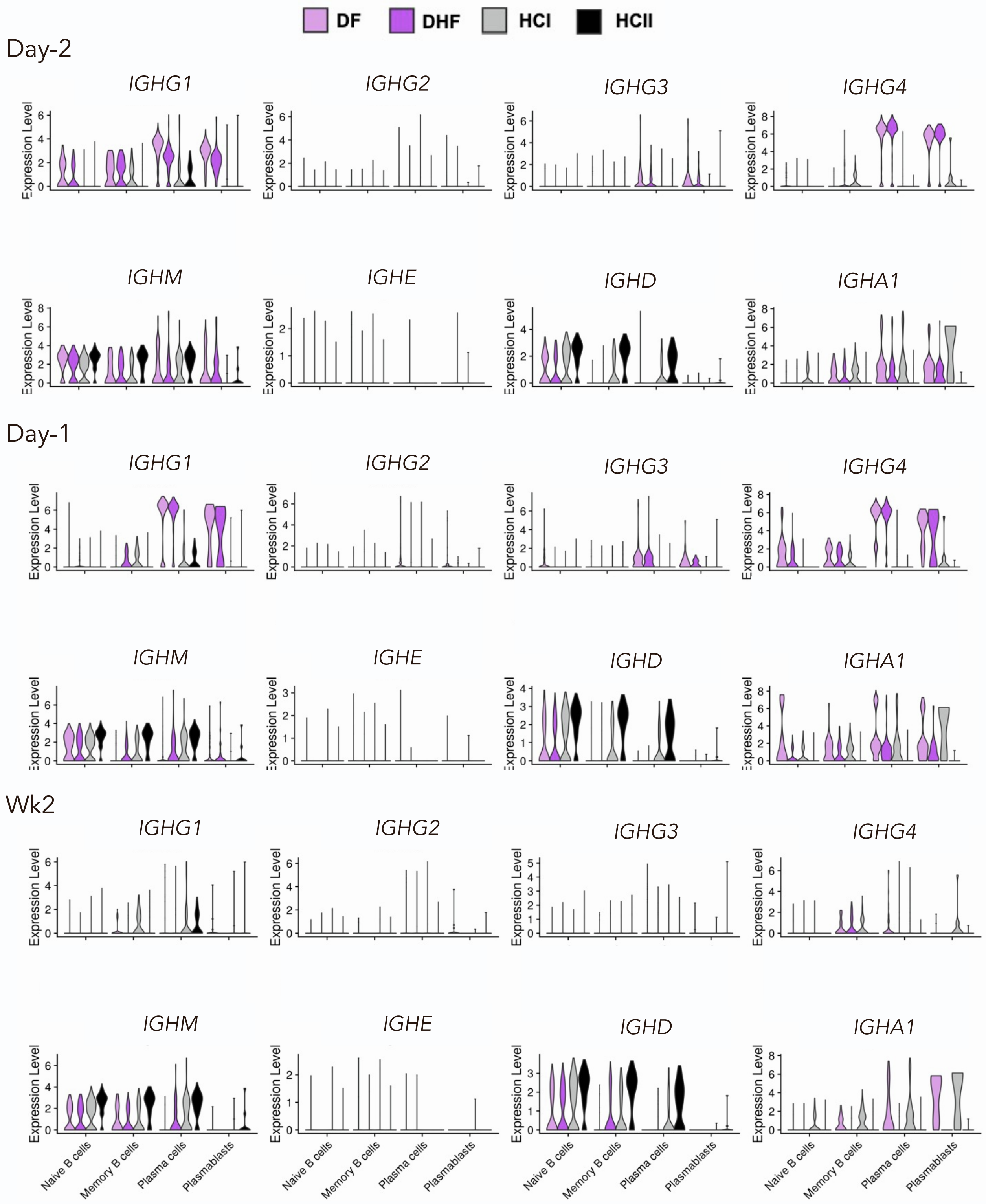

Figure S20. Violin plots representing the normalised expression of Ig genes (*IGHG1*, *IGHG2*, *IGHG3*, *IGHG4*,*IGHM* and *IGHA1*) in the DF and DHF patients at Day-2, Def, and Wk2. Related to Figure 5B.

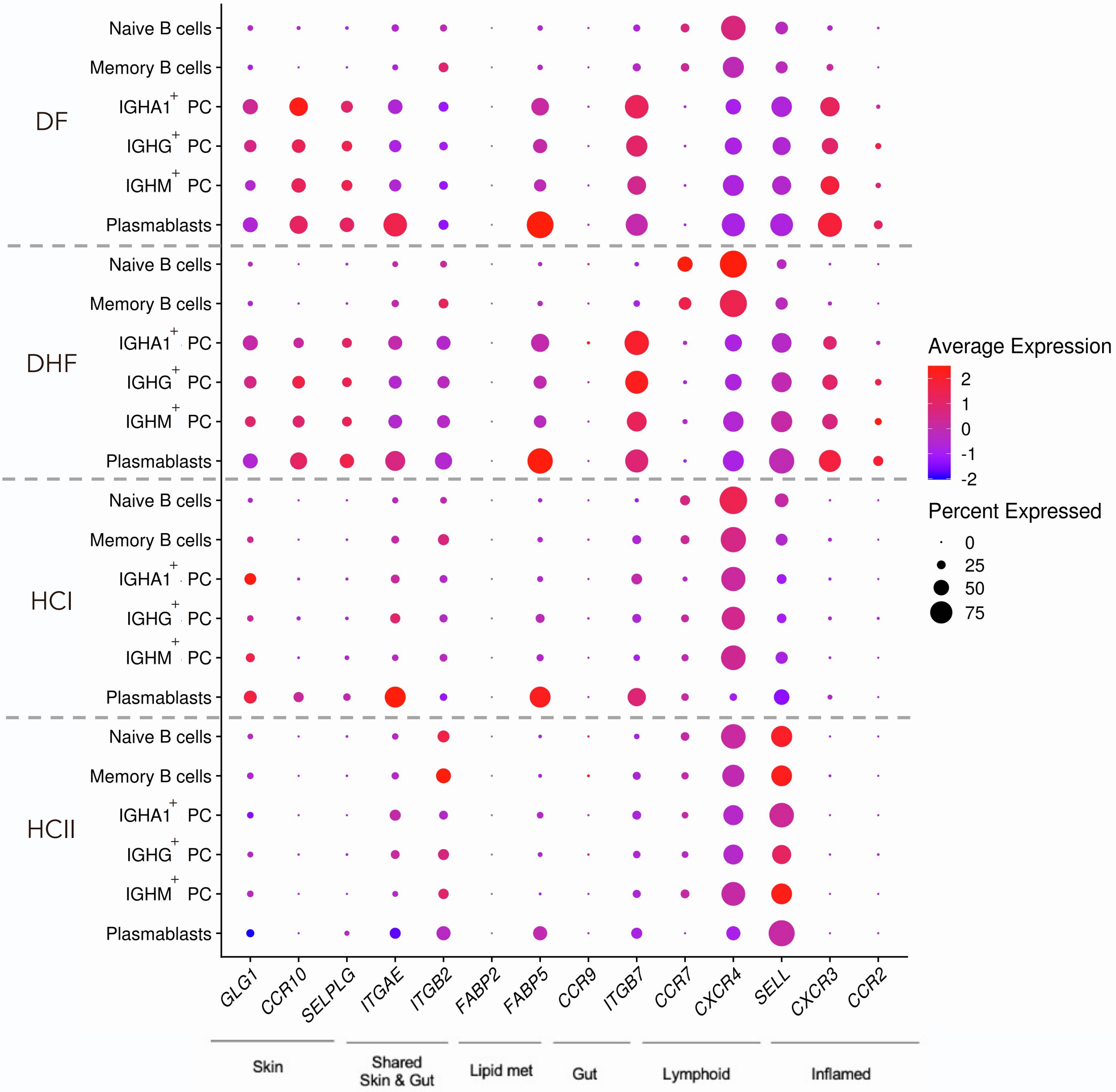

Figure S21. Dotplots representing the average gene expression of the tissue-homing genes of interest. The dot sizes represent the proportion of cells expressing the genes. Related to Figure 5D.

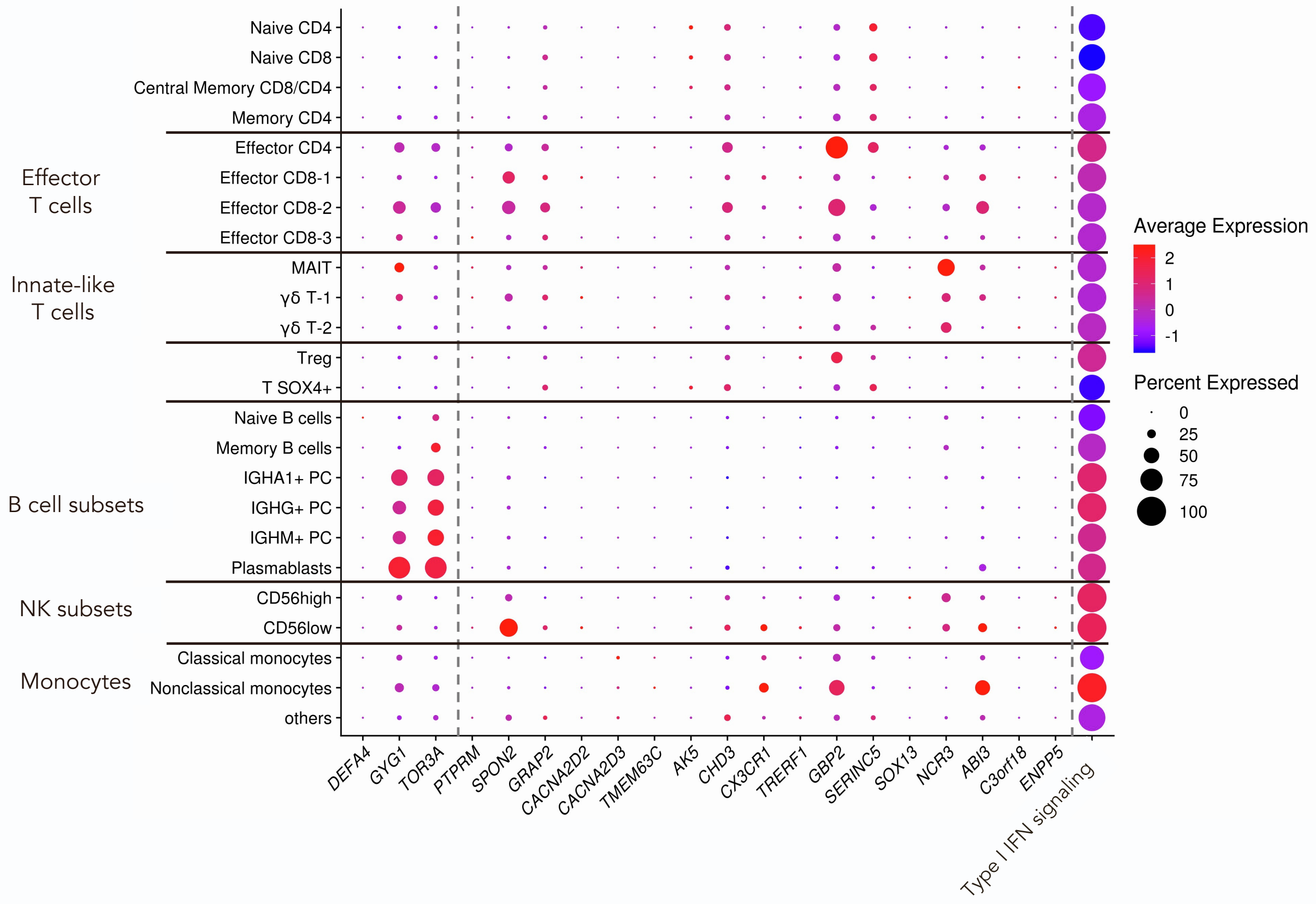

Figure S22. Dotplots representing average gene expression based on scRNA-seq in this study, of the 20 severe-dengue-predictive genes as proposed by Robinson and colleagues (Robinson et al. 2019), together with the HVGs that are associated in Type I IFN signaling across immune subpopulations. Three genes (left) are over-expressed whereas 17 are under-expressed in the DHF/DSS group, compared to the DF group (Robinson et al. 2019). The dot sizes represent the proportions of cells in particular populations that express the genes of interest. Related to Figure 3 and 5.

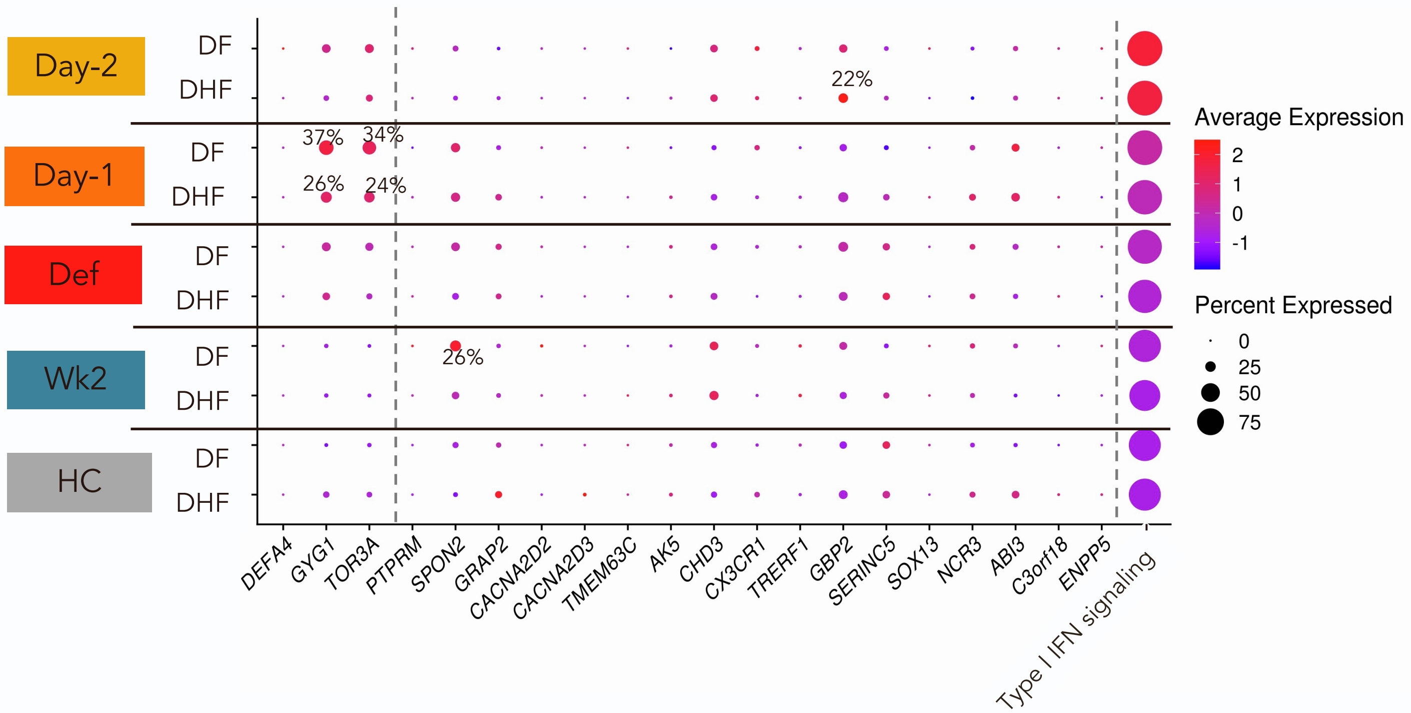

Figure S23. Dotplots representing average gene expression based on scRNA-seq in this study, of the 20 severe-dengue-predictive genes as proposed by Robinson and colleagues (Robinson et al. 2019), together with the HVGs that are associated in Type I IFN signaling over the course of DENV infection. Three genes (left) are over-expressed whereas 17 are under-expressed in the DHF/DSS group, compared to the DF group (Robinson et al. 2019). The dot sizes represent the proportions of cells in particular populations that express the genes of interest. Related to Figure 3 and 5.

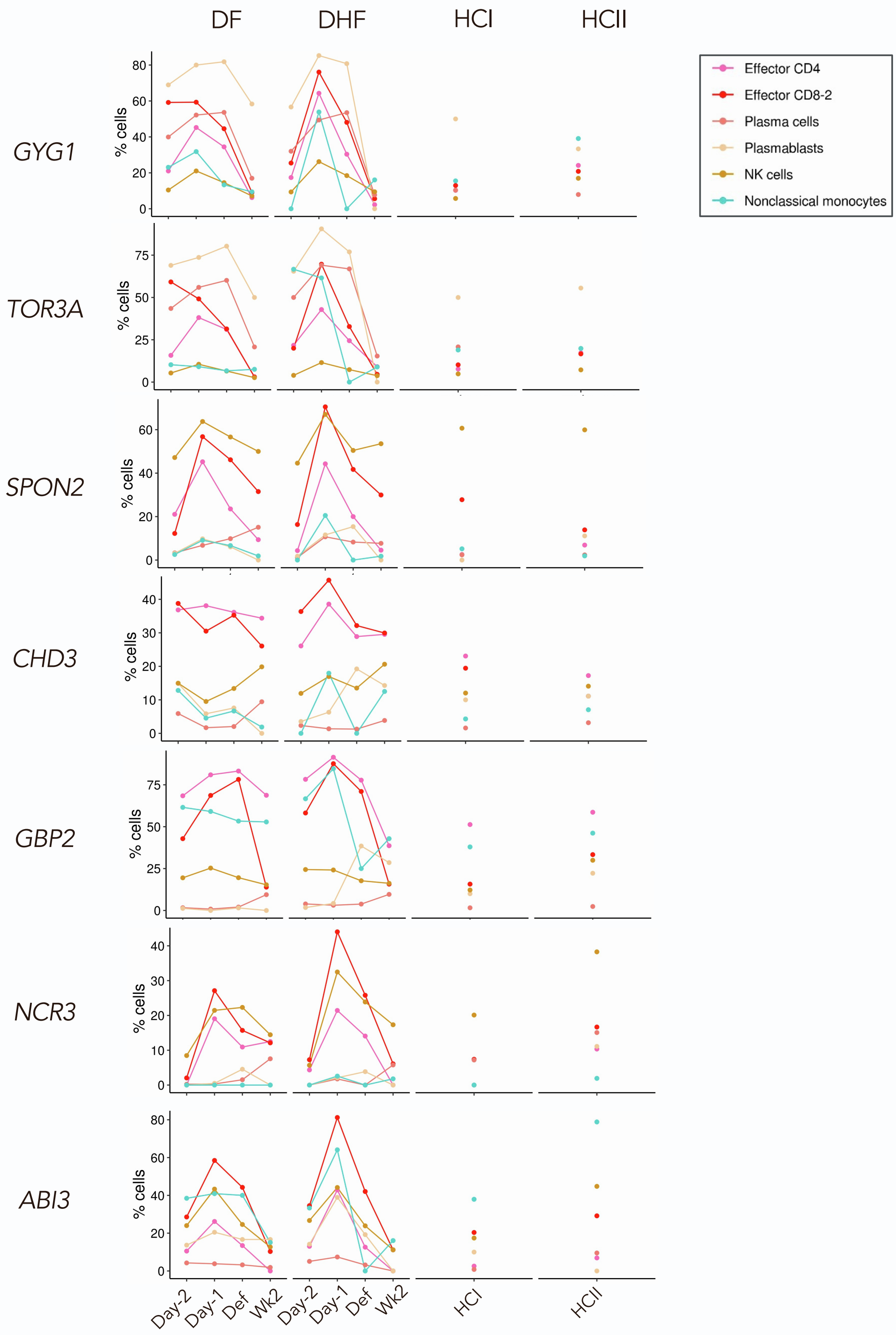

Figure S24. Line plots showing the percentages of cells expressing severe-dengue-predictive genes of interest (Robinson et al. 2019) across the four timepoints in our DF and DHF patients. Related to Figure 3 and 5.

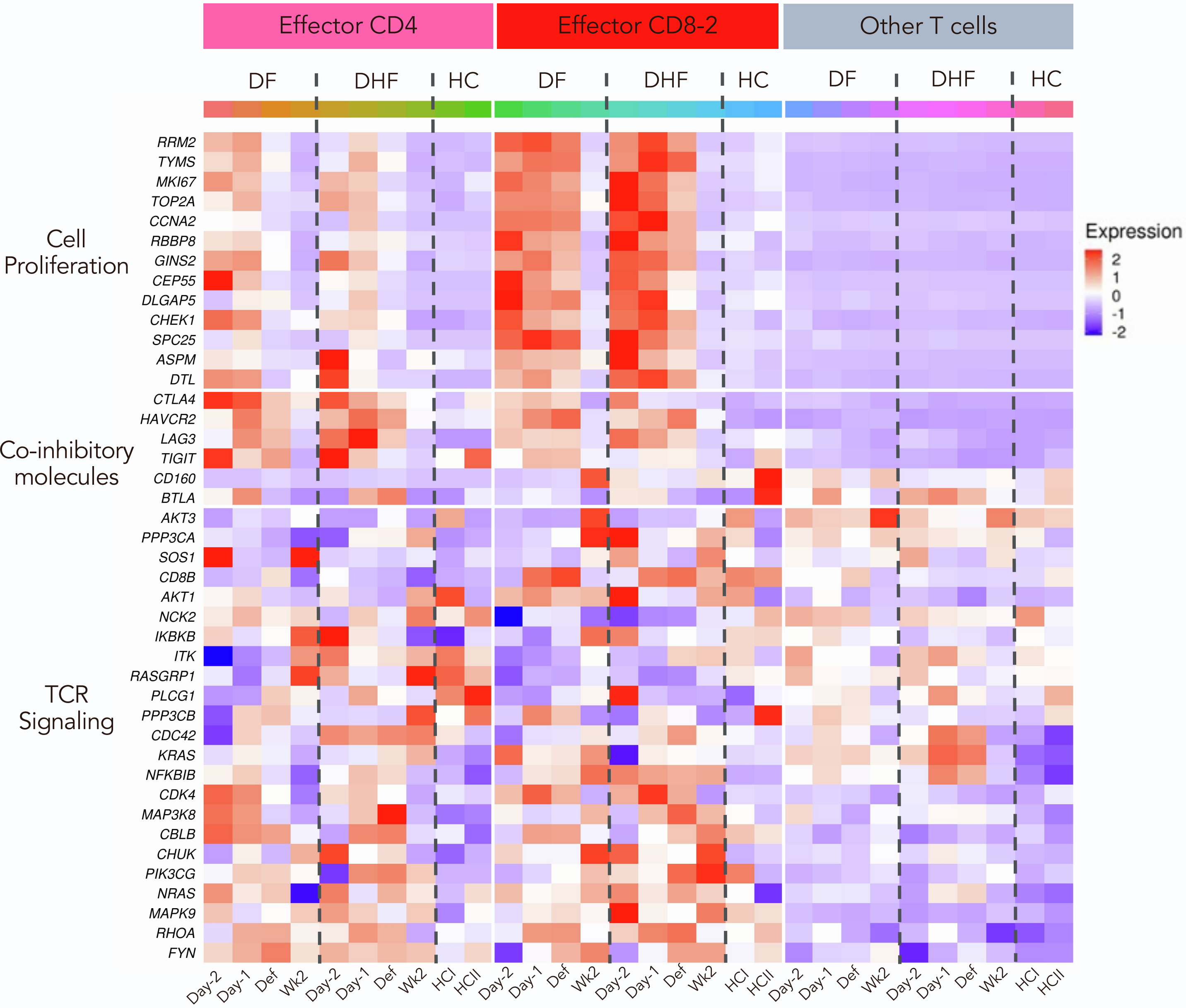

Figure S25. Heatmap representing the relative expression of genes that are associated with cell proliferation, co-inhibitory molecules, and TCR signaling functions, as proposed by Chandele and coworkers (Chandele et al. 2016). Related to Figure 3.
